# Snail collaborates with EGR-1 and SP-1 to directly activate transcription of MMP 9 and ZEB1

**DOI:** 10.1038/s41598-017-18101-7

**Published:** 2017-12-19

**Authors:** Wen-Sheng Wu, Ren-In You, Chuan-Chu Cheng, Ming-Che Lee, Teng-Yi Lin, Chi-Tan Hu

**Affiliations:** 1 Institute of medical biotechnology, college of Medicine, Tzu Chi University, Hualein, Taiwan; 2 Department of Surgery, Buddhist Tzu Chi General Hospital, School of Medicine, Tzu Chi University, Hualien, Taiwan; 3 Department of Laboratory Medicine, Hualien Tzu Chi Hospital, Buddhist Tzu Chi Medical Foundation, Hualien, Taiwan; 4 Research Centre for Hepatology, Department of Internal Medicine, Buddhist Tzu Chi General Hospital and Tzu Chi University, Hualien, Taiwan

## Abstract

The Snail transcription factor plays as a master regulator of epithelial mesenchymal transition (EMT), one of the steps of tumor metastasis. Snail enhances expressions of a lot of mesenchymal genes including the matrix degradation enzyme matrix metalloproteinases 9 (MMP9) and the EMT transcription factor zinc finger E-box binding homeobox 1 (ZEB1), however, the underlying mechanisms are not clarified. Herein, we investigated how Snail upregulated transcription of ZEB1 and MMP9 induced by the tumor promoter 12-O-tetradecanoyl-phorbol 13-acetate (TPA) in hepatoma cell HepG2. According to deletion mapping and site directed mutagenesis analysis, the TPA-responsive elements on both MMP9 and ZEB1 promoters locate on a putative EGR1 and SP1 overlapping region coupled with an upstream proposed Snail binding motif TCACA. Consistently, chromatin immunoprecipitation (ChIP) assay showed TPA triggered binding of Snail, EGR1 and SP1 on MMP9 and ZEB1 promoters. Double ChIP further indicated TPA induced association of Snail with EGR1 and SP1 on both promoters. Also, electrophoresis mobility shift assay revealed TPA enhanced binding of Snail with a MMP9 promoter fragment. According to shRNA techniques, Snail was essential for gene expression of both ZEB1 and MMP9. In conclusion, Snail transactivates genes involved in tumor progression *via* direct binding to a specific promoter region.

## Introduction

The poor prognosis of hepatocellular carcinoma (HCC), one of the most devastating cancers worldwide, is due to frequent recurrence and metastasis after surgical resection^1^. Tumor metastasis occurs via complicated processes, including epithelial mesenchymal transition (EMT), migration and invasion of primary tumor, followed by intravasation, extravasation and colonization at the metastatic loci^2^. In the past decades, Snail was highlighted as one of the transcription factors responsible for tumor progression^3–5^. Specifically, over-expression of Snail was found to be associated with poor prognosis of HCC^5,6^ and may accelerate EMT and invasion of HCC^2,6^. Also, silencing of Snail effectively suppressed tumor growth and invasiveness of HCC^7^.

Conventionally, Snail was well known to be a negative regulator of gene expression responsible for diverse cellular effects^8^. Snail plays as one of the essential EMT transcription factor^9^ typically repressing the transcription of E-cadherin, one of the essential epithelial markers^10,11^. On the other hand, the role of Snail as a transcriptional activator was also greatly implicated. Snail may enhance mesenchymal markers including fibronectin, collagens, the matrix degradation enzyme matrix metalloproteinases 2 and 9 (MMP2 and MMP9) and other EMT transcription factors such as Twist and zinc finger E-box binding homeobox 1 (ZEB1)^8,12^. Among them, MMP9^13–15^ and ZEB1^16,17^ were known to be involved in HCC progression. Moreover, Snail may enhance transcription of MMP9 in HCC^18^.

The transcriptional mechanisms for Snail to suppress E-cadherin have been well elucidated^9^. Snail contains tandem Cys_2_-His_2_ zinc-finger motifs in the C-terminal capable of binding to the E-boxes (5′-CACCTG) on E-cadherin promoter, interfering with its transcription. This was ascribed to the Snail-triggered recruitment of various epigenetic machineries such as histone deacetylase and Polycomb repressive complex 2 to the E-cadherin promoter resulting in histone H3K4 deacetylation and H3K27 methylation^19–21^. Together, these chromatin-modifying enzymes function in a Snail-mediated, highly orchestrated fashion to suppress E-cadherin transcription. In contrast, the detailed mechanisms for Snail to upregulate transcription of genes such as MMP9 and ZEB1 are far less clarified. Previous studies indicated that Snail activated transcription of MMP9 and ZEB1 indirectly *via* regulation of other transcription factors such as Twist, Ets-1 and SP1 or microRNAs (see discussion, section 1). However, the possibility that Snail directly activates target gene promoter was also highlighted recently. Rembold *et al*. showed that Snail positively modulated transcriptional activation of target genes involved in Drosophila development *via* direct binding to promoters^22^. Our recent study also indicated that Snail, in collaboration with EGR1 and SP1, may directly activate transcription of the inhibitor of cyclin-dependent kinase 4/6 (CDK4/6), p15^INK4b^, in HepG2 cell stimulated by the phorbol ester tumor promoter 12-O-tetradecanoyl-phorbol 13-acetate (TPA)^23^. Moreover, we pinpointed a potential consensus Snail binding motif (TCACA) upstream of EGR/SP1 overlapping region on p15^INK4b^ promoter^23^. Interestingly, the same sequence architecture was also found within promoters of a lot of Snail-upregulated genes including MMP9 and ZEB1 (Fig. S1A and B). It is tempting to investigate whether there is consensus mechanism for Snail to upregulate gene expression *via* direct binding to specific promoter region.

In this report, we did find TPA induced gene expression of ZEB1 and MMP9 by the similar transcriptional mechanism as that of p15^INK4b^. Specifically, the direct binding of Snail toward the consensus region was shown.

## Results

### TPA induced gene expression of ZEB1 and MMP9 in HepG2 and HCC340 cells

**Figure 1:**
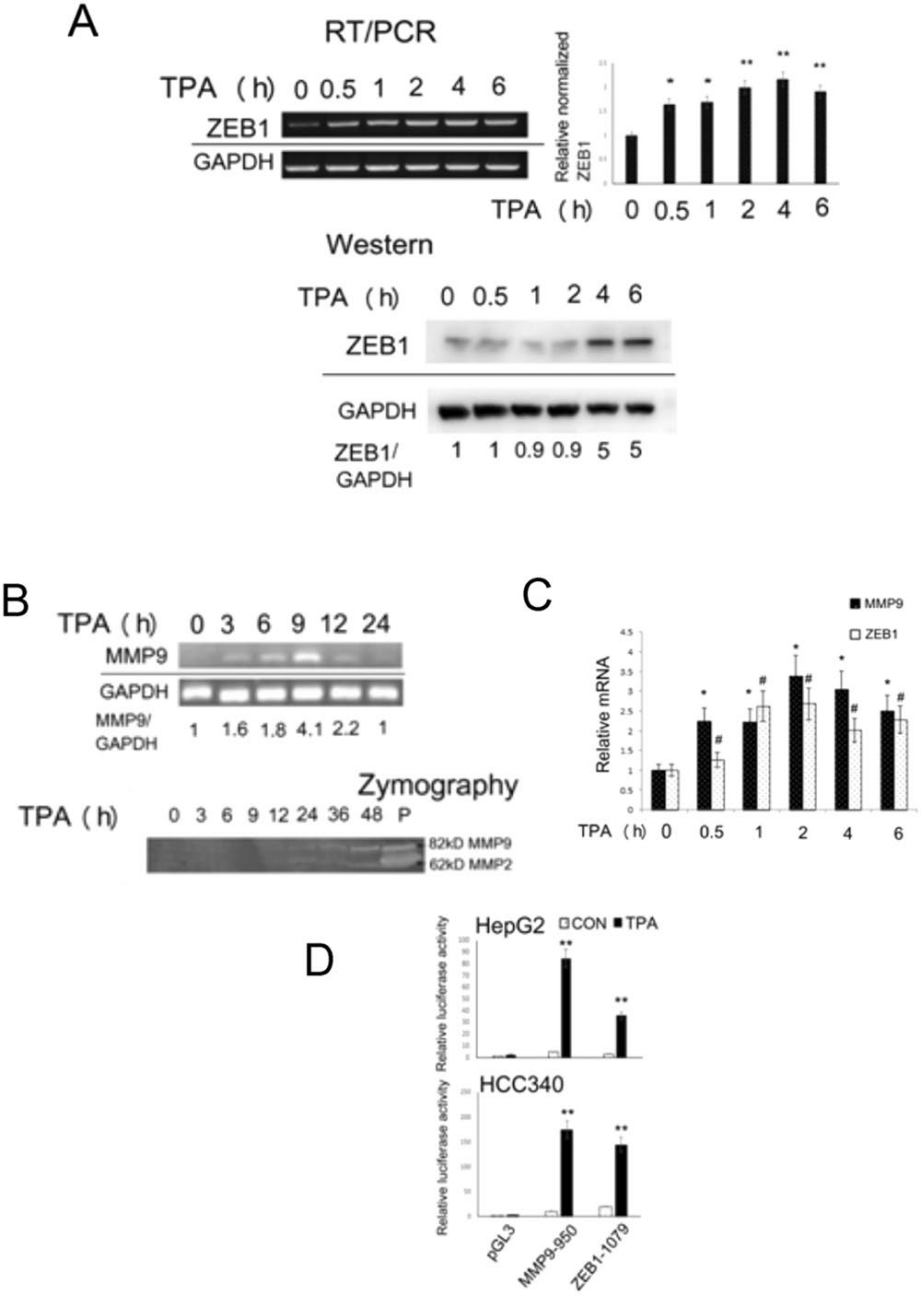
TPA induced gene expression of MMP9 and ZEB1. HepG2 cells (A,B,C and D upper panel) and HCC340 (D lower panel) were treated with 50 nM TPA for indicated times (**A**,**B**,**C**) or 12 h (**D**). RT-PCR of ZEB1 (A, upper panel) and MMP9 (B, upper panel), quantitative RT-PCR of ZEB1and MMP9 (**C**), Western blot of ZEB1 (A, lower panel) and dual luciferase promoter assay of MMP9-950 and ZEB1-1079 (containing full length promoters of MMP9 and ZEB1) (**D**), were performed. In the upper panel of (A), relative ZEB1 cDNA (normalized with GAPDH) was quantitated, taking the data of time zero as 1.0. (**^,^*) represent the statistical significant difference (p < 0.005, p < 0.05, N = 3) of ZEB1 cDNA between the indicated samples and the time zero group. In lower panel of (A) and upper panel of (B), the numbers below the figure are the relative ZEB1 protein and MMP9 cDNA (normalized with GAPDH), respectively, taken the data of time zero as 1.0. The data are average of three reproducible experiments with coefficient of variation (C.V) of 8.0%. Lower panel of B demonstrated the Zymography of active MMP9 (82kD) and MMP2 (62kD) secreted into the conditioned mediums of TPA-treated HepG2 collected at the time indicated. The molecular weights of MMP9 and MMP2 are verified by respective MMP standards denoted as “P” in the most right lane. The figure is representative of two reproducible experiments. In (**C**), relative mRNA was calculated in the quantitative real time RT-PCR analysis, taking the data of time zero as 1.0. (*) and (#) represent the statistically significant difference (p < 0. 05, N = 3) of relative mRNA of MMP9 and ZEB1 between each of the TPA-treated samples and time zero group. In (**D**), the relative activity of each promoter was quantitated, taking the data of pGL3 as 1.0. (**) represent the statistical significant difference (p < 0.005, N = 4) of relative promoter activity between each of the TPA-treated samples and untreated group.

Initially, we analyzed the time course of TPA-induced gene expression of ZEB1 in HepG2 by RT-PCR. Treatment of the cells with TPA for 0.5–1 h elevated ZEB1 mRNA by 1.6~1.7-fold, which increased by 2.0~2.3-fold within 2–6 h (Fig. 1A upper panel). Quantitative real time RT-PCR also showed the TPA-induced elevation of ZEB1 mRNA by 1.28-fold at 0.5 h followed by 2.0~2.7-fold within 1–6 h (Fig. 1C). Western blot analysis showed that TPA induced ZEB1 protein expression at 4 and 6 h by 5.0-fold (Fig. 1A lower panel). On the other hand, RT-PCR showed slight induction of MMP9 mRNA induced by TPA at at 3 and 6 h, which further increased by 4.1-fold at 9 h and declined to basal level until 24 h (Fig. 1B, upper panel). Quantitative RT-PCR also showed the TPA-induced elevation of MMP9 mRNA by 2.2-fold within 0.5~1 h and 2.5~3.4-fold within 2~6 h (Fig. 1C). By zymography, TPA induced minor secretion of active MMP9 (82 kD) into HepG2 conditioned medium at 24 and 36 h, which further greatly increased at 48 h (Fig. 1B, lower panel). In contrast, the TPA-induced secretion of MMP2 (62 kD) was less prominent during this period. Thus gene expression of both MMP9 and ZEB1 can be induced by TPA.

### TPA induced transcriptional activation of ZEB1 and MMP9 in HepG2 and HCC340 cells

Whether TPA-induced gene expression of ZEB1 and MMP9 can be associated with promoter activation was further investigated by promoter assay using pGL3 repoter plasmid. As shown in Fig. 1D, TPA induced activation of promoter construct MMP9-950 (the full length MMP9 promoter, 950 bp upstream of translational start site) and ZEB1-1079 (full length ZEB1 promoter, 1079 bp upstream of translational start site) by 17.5- and 6.0-fold, respectively in HepG2 (Fig. 1D, upper panel). Moreover, TPA can induce activation of MMP9-950 and ZEB1-1079 by 35.0- and 7.0-fold, respectively, in HCC340, a patient derived HCC cell line^24^ (Fig. 1D, lower panel).

### Deletion mapping for identification of the TPA-responsive element on MMP9 promoter

**Figure 2:**
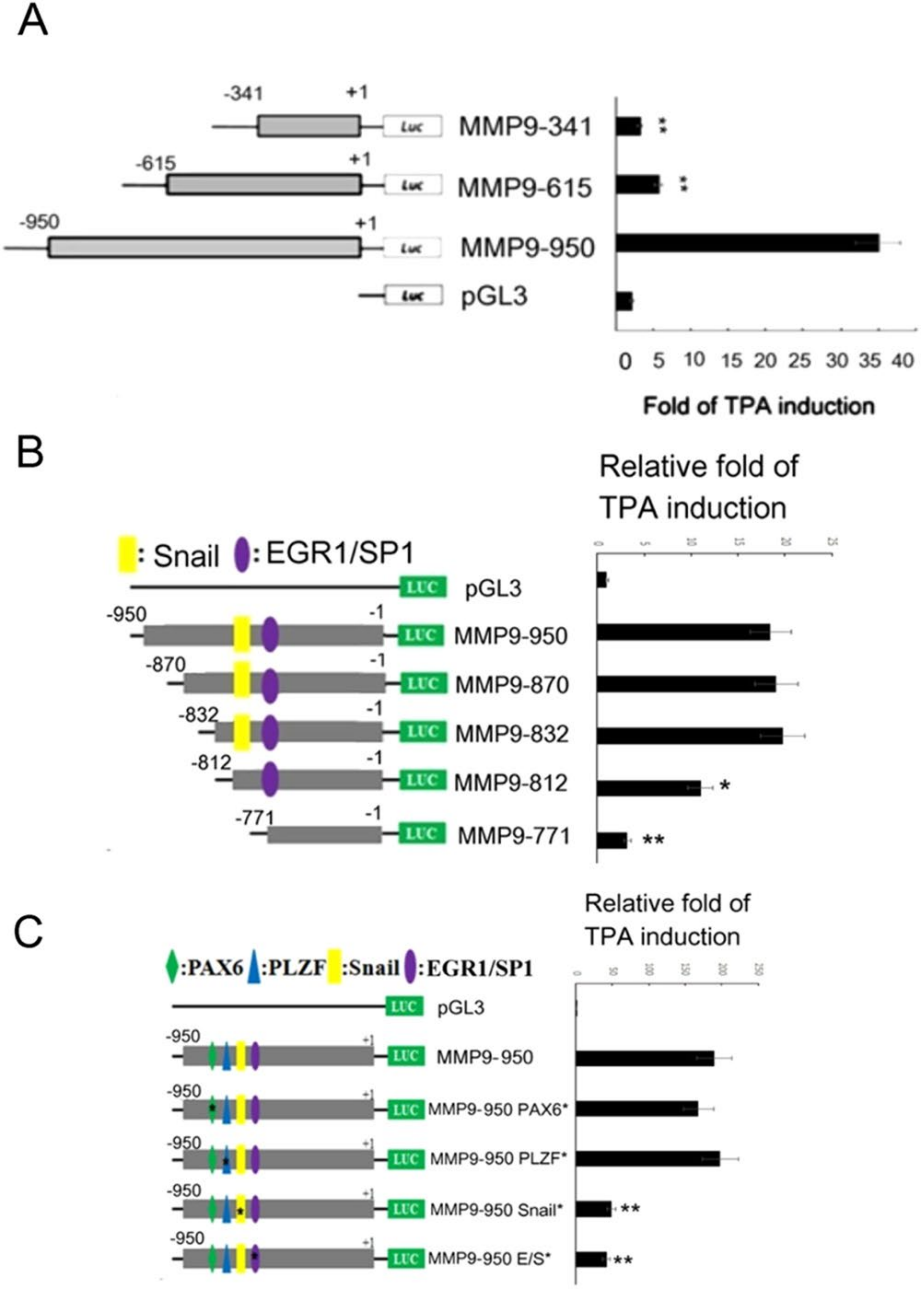
Deletion mapping and mutagenesis analysis for TPA-responsive element in MMP9 promoter. HepG2 cells were transfected with pGL3 vector, the indicated MMP9 promoter plasmids including full length promoter (MMP9-950) (**A**,**B**,**C**), various 5′ deleted promoters (**A**,**B**), or mutant promoters with alteration on the putative binding region of indicated transcriptional factors (**C**) for 24 h. Subsequently, the cells were untreated or treated with 50 nM TPA for 12 h and then dual luciferase assay were performed. The relative fold of TPA induction for each promoter were quantitated as the activity of TPA treated *vs* untreated (demonstrated on the right panel), taking the data of pGL3 as 1.0. (**^,^*) represent the statistically significant difference (p < 0.005, p < 0.05, respectively, N = 4) of relative TPA-induced promoter activity between each of the indicated samples and the full length promoter (MMP9-950) group.

To identify the TPA-responsive region on MMP9 promoter, deletion mapping using full length MMP9 promoters coupled with shorter promoter constructs with deletions on the 5′ end (Fig. 2A, left panel) was performed in HepG2. The full length MMP9 promoter MMP9-950 and the other two 5′-deletion constructs MMP9-615 and MMP9-341, containing MMP9 promoter fragments of 615 and 341 bp respectively, were introduced into HepG2 for 24 h, followed by treatment with TPA for 12 h. The TPA-induced activation of MMP9-950 increased by 17-fold as compared with that of pGL3 vector (Fig. 2A, right panel). Remarkably, the TPA-induced activation of MMP9-615 and MMP9-341 were only 2.3- and 1.5-fold, respectively, much less than that of MMP9-950 (Fig. 2A, right panel). This indicated that the major TPA-responsive region on MMP9 promoter locates between 950 bp to 615 bp upstream of the transcriptional initiation site. To further dissect the exact TPA-responsive element, four more detailed 5′-deletion constructs MMP9-870, MMP9-832, MMP9-812 and MMP9-771 containing MMP9 promoter fragments of 870, 832, 812 and 771 bp, respectively, were employed. As shown in Fig. 2B, TPA-induced promoter activation of MMP9-870 and MMP9-832 exhibited no significant difference from that of MMP9-950, whereas those of MMP9-812 and MMP9-771 decreased by 40 and 82%, respectively, as compared with that of MMP9-950. Thus the TPA-responsive element on MMP9 appeared to locate between 832 bp and 771 bp upstream of the transcriptional initiation site.

### Proposed Snail binding motif and putative EGR1/SP1 regions were required for TPA-induced MMP9 promoter activation

According to Genomatix software, there are EGR1/SP1 overlapping region agccccccACCCcccg (The putative regions of EGR1 and SP1 are: EGR1: 5′aagagccccccACCCccg 3′, SP1: 5′agccccCCACcccccgt3′) locating downstream of the proposed Snail binding motif TCACA^23^ within the TPA-responsive element (−832 to −771 bp) on MMP9 promoter (Fig. S1A). This sequence architecture is very similar to that of the TPA-responsive regions on p15^INK4b^ promoter identified in our previous study^23^. To investigate whether they are also essential for TPA-induced MMP9 promoter activation, MMP9-950 mutants with altered proposed Snail binding motif and the EGR/SP1 overlapping region, denoted as MMP9-950 Snail * and MMP9-950 E/S*, respectively, were obtained by site directed mutagenesis. We examined whether TPA-induced activation of these mutant promoters decreased as compared with the wild type promoter. To confirm the specificity of these regions, MMP9-950 mutants with alteration in two nearby transcription factor binding motifs PAX6 and PLZF denoted as MMP9-950 PAX* and MMP9-950 PLZF*, respectively, were also included. As shown in Fig. 2C, the TPA-induced activation of MMP9-950 Snail* and MMP9-950 E/S* decreased by 74 and 78%, respectively, compared with that of wild type MMP9-950. In contrast, no significant change of TPA-induced promoter activation of MMP9-950 PAX6* and MMP9-950 PLZF* were observed. We noticed that MMP9-950 Snail*, with mutation of the Snail binding motif, exhibited more significant reduction (Fig. 2B) than MMP9-812 with deletion of Snail binding motif (Fig. 2C) (75% *vs* 40%). To address this issue, we analyzed the TPA-induced activity of MMP9-812 and MMP9-950 Snail* simultaneously in a single promoter assay. As shown in Fig. S3, TPA-induced promoter activity of MMP9-812 and MMP9-950 Snail* decreased by 58 and 64%, respectively, in comparison with that of full length MMP9-950. Thus, the reduction of TPA-induced activity of these two mutants appeared more comparable in a single (Fig. S3) than in separate (Fig. 2B and C) assay. Taken together, EGR1/SP1 overlapping region coupled with the upstream proposed Snail binding motif was specifically required for TPA-induced activation of MMP9 promoter.

### TPA induced binding of Snail and EGR1/SP1 on MMP9 promoter

**Figure 3:**
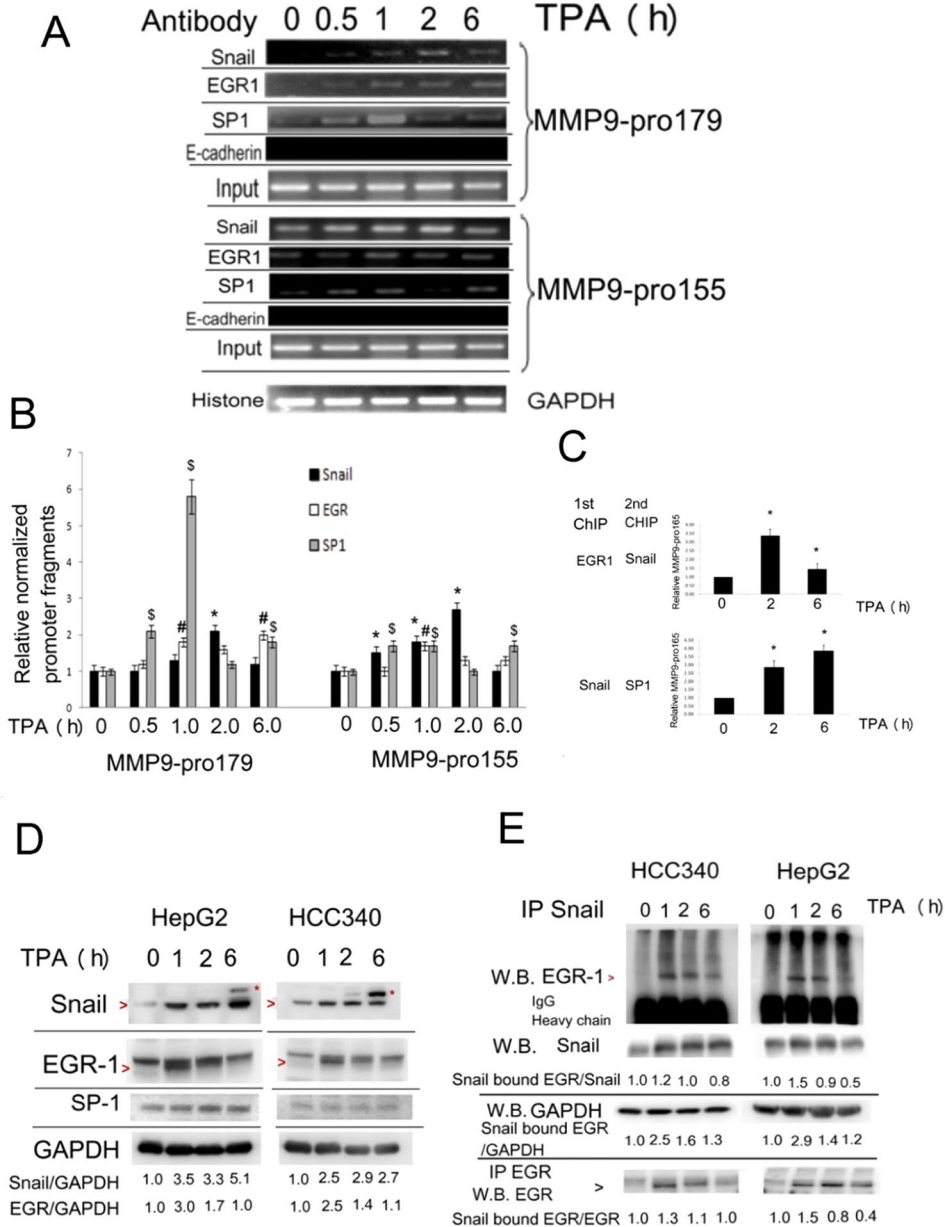
TPA induced binding of Snail, EGR and SP1 on MMP9 promoter. HepG2 cells were treated with 50 nM TPA for the times indicated, ChIP assay for binding of Snail, EGR1 and SP1 on MMP9-pro179 and MMP9-pro155 (**A**,**B**) and double ChIP of EGR (1^st^)/Snail (2^nd^) and Snail (1^st^)/SP1 (2^nd^) for amplifying MMP9-pro165 using quantitative PCR (**C**), were performed. In (**A**), ChIPs of histone binding on GAPDH promoter and E-cadherin on MMP9 promoter were used for positive and negative control, respectively. Input is for loading control for ChIP. (**B**) is the quantitative figure for (**A**). Relative amount of the indicated promoter fragments was normalized with the binding amount of histone3 on GAPDH promoter, taking the time zero group as 1.0. (*^, #^ and ^$^) represent statistic significance (p < 0.05, N = 3) between the indicated sample with the time zero group. In (**C**), (**^,^*) represent the statistically significant difference (p < 0.005, p < 0.05, respectively, N = 3) of relative MMP9-pro165 between each of the indicated samples and time zero group. Schematic maps of MMP9-pro179, MMP9-pro165 and MMP9-pro155 across the indicated regions on MMP9 promoter are demonstrated in supplemental Fig. 2A. (**D**), (**E**). HepG2 cells were treated with 50 nM TPA as indicated, Western (D) and IP/Western (**E**) blot of indicated proteins were performed. In (D), “>” indicated the position of Snail and EGR-1, whereas * indicated the un-identified protein with M.W. slightly larger than Snail. In (**D**), (**E**) the numbers below are relative intensities of indicated protein or Snail bound EGR1 *vs* GADPH as a loading control, and “Snail bound EGR1” *vs* immunoprecipitated Snail or EGR1as an input, taking the time zero sample as 1.0. The data were average of two reproducible experiments with C.V. of 0.05. In (E), I.P.: Immunoprecipitation, W.B.:Western blot. The lines between the images in (**A**), (**D**) and (**E**) are included for dividing gels of different ChIP assays, Western blot or IP, respectively, of indicated molecules.

We further examined whether TPA may trigger binding of Snail, EGR1 and SP1 on MMP9 promoter in HepG2 by ChIP assay. The MMP9 promoter fragment MMP9-pro179 (−865~−685 bp) containing EGR1/SP1 overlapping region, coupled with the upstream proposed Snail binding motif (Fig. S2A), were PCR-amplified from chromatins precipitated by Snail Ab. As shown in Fig. 3A and B, after TPA treatment, binding of Snail on MMP9-pro179 was scarcely observed at 0.5 and 1 h, reached the peak by 2.5-fold at 2 h followed by a decrease at 6 h. On the other hand, ChIP of EGR1 showed the TPA-induced binding of EGR1 on MMP9-pro179 by 1.8-fold at 1 h, which sustained until 6 h. Also, ChIP of SP1 revealed that the TPA-induced binding of SP1 on MMP9-pro179 increased at 0.5 and 1 h by 2.1 and 5.8-fold, respectively, followed by a decrease at 6 h.

To confirm whether TPA may induce binding of Snail specifically on the proposed Snail binding motif, ChIP primer was designed for amplifying the region between −955 and −800 (MMP9-pro155) containing the proposed Snail binding motif (TCACA) but not EGR/SP1 overlapping region (Fig. S2A). As shown in Fig. 3A and B, the TPA-induced binding of Snail on MMP9-pro155 increased by 1.5-1.8 fold within 0.5–1 h, reached the peak by 2.7-fold at 2 h, followed by a significant decrease at 6 h. On the other hand, TPA also induced significant binding of EGR1 on MMP9-155 by 1.2–1.7-fold during 1–6 h (Fig. 3A). In addition, TPA induced binding of SP1 on MMP9-155 at 0.5 and 1 h, which abolished at 2 h and increased again at 6 h, revealing a biphasic induction.

### TPA induced co-localization of Snail with EGR and SP1 on MMP9 promoter

Given that TPA induced binding of Snail, EGR1 and SP1 on both MMP9-pro179 and MMP9-pro155 which contain the region around Snail binding motif and the nearby EGR1/SP1 overlapping region, it is tempting to prove that TPA may trigger co-localization of Snail with EGR1 and/or SP1 on MMP9 promoter. To address this issue, double ChIP of Snail coupled with EGR1 or SP1 was performed using real time PCR for quantitation. The double ChIP primer was designed for amplifying the fragment between −820 and −655 bp (MMP9-pro165) containing both the proposed Snail binding motif TCACA and EGR/SP1 overlapping region (S2A Fig). EGR1 Ab was used for the 1^st^ ChIP and Snail Ab for the 2^nd^ to detect whether Snail co-localized with EGR1 on this promoter fragment. As shown in Fig. 3C, treatment of the cells with TPA for 2 h increased MMP9-pro165 (amplified from EGR1/Snail double ChIP) by 3.5-fold, which then declined by 1.5-fold at 6 h. Also, Snail Ab was used for the 1^st^ ChIP and SP1 Ab for the 2^nd^ to detect whether Snail co-localized with SP1 on this promoter fragment. Treatment of TPA increased MMP9-pro165 (amplified from Snail/SP1 double ChIP) by 2.86-fold at 2 h and further increased by 3.86-fold at 6 h. Collectively, these results indicated that TPA can induce co-localization of Snail with EGR1 and SP1 on MMP9 promoter.

According to double ChIP as described above, there is great possibility that these transcription factors interact with each other upon TPA induction. Our previous report^23^ has suggested TPA can induce not only gene expression of Snail, EGR-1 and SP-1 but also association between Snail and EGR1 or EGR1 and SP1 in HepG2. We further investigated these molecular events in a more comprehensive manner in both HepG2 and HCC340. As shown in Fig. 3D, TPA induced expression of Snail at 1–2 h by 3.3~3.5-fold and further increased by 5.1-fold at 6 h in HepG2, whereas it induced expression of Snail at 1–2 h by 2.5~2.9-fold, and sustained until 6 h in HCC340. Notably, an un-identified band (indicated by*) with a molecular weight slightly higher than Snail at 6 h time point was observed in both cells. On the other hand, TPA induced significant expression of EGR1 (as the band indicated by >) by 2.5~2.9-fold at 1 h and declined thereafter in both cells. In addition, TPA marginally induced SP1 at 2 h in HepG2 but not in HCC340. We also investigated whether TPA-induced Snail and EGR was controlled on the transcriptional level. To do this, actinomycin D, an inhibitor of RNA polymerase was employed. As shown in Fig. S4, co-treatment of 20 nM actinomycin D with TPA reduced the TPA-induced Snail and EGR1 in HCC340 by 80%, Thus induction of both Snail and EGR1 by TPA are transcription-dependent in HCC340. Although actinomycin D prevented TPA-induced EGR1 by 90% in HepG2, it cannot suppress TPA-induced Snail expression (Fig. S4). In fact, actinomycin D even greatly enhanced TPA-induced Snail expression, the underlying mechanism remained elusive Thus how Snail was induced by TPA in HepG2 needs to be clarified further.

To analyze protein–protein interaction, IP of Snail followed by Western of EGR1 was performed in both cells. Treatment of HepG2 and HCC340 by TPA for 1 h triggered the association of Snail with EGR1 by 2.9 and 2.5-fold, respectively, which slightly declined at 2 h and abolished at 6 h, using input of GAPDH as a loading control (Fig. 3E, see the ratio of Snail bound EGR/GAPDH). However, the increase of relative association of Snail with EGR1 is less prominent at 1 h (1.2–1.5-fold) in HepG2 and HCC340, if the data was normalized with Western of Snail from the immunoprecipitate of Snail, or Western of EGR1 from the immunoprecipitate of EGR1 (Fig. 3E, see the ratio of Snail bound EGR/Snail and Snail bound EGR/EGR). This was obviously due to that both Snail and EGR1 were elevated by TPA within this period (Fig. 3D,E). Nevertheless, the total association between Snail with EGR1 was indeed increased as their amount increased.

In addition, we observed whether TPA also induces the association of Snail with SP1. Although we found TPA induce significant SP1 expression as revealed in the IP of SP1coupled with Western blot of SP1 at 2 h (Fig. S6), IP of SP1coupled with Western blot of Snail showed marginal increase of association of SP1 and Snail at 2 h compared with the time zero group in HepG2 (Fig. S6).

### Snail binds with specific MMP9 promoter fragment *in vitro*

**Figure 4:**
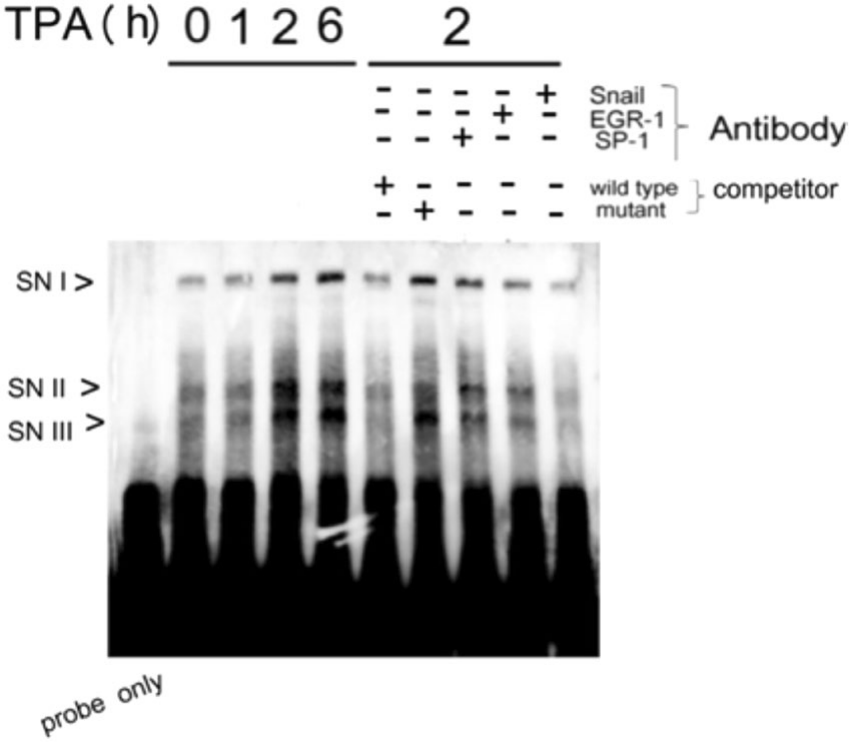
EMSA for *in vitro* binding of Snail to the proposed Snail binding region on MMP9 promoter. Nuclear extracts obtained from HepG2 were treated with 50 nM TPA for the times indicated, followed by EMSA using MMP9-proSN (lanes 2-5). Unlabed wild-type or mutant competitors in 200X amount were included in the EMSA for HepG2 treated with TPA for 2 h (lanes 6 and 7). Lane 1 is the sample of probe only. For antibody blocking analysis, each of the indicated antibody was preincubated with the nuclear extract from HepG2 treated with TPA for 2 hr followed by EMSA reaction (lanes 8–10). Schematic map of the EMSA probes (MMP9-porSN), and the competitor (MMP9-porSN) located around Snail binding motif upstream of the EGR-1/SP1 overlapping region on MMP9 promoter is demonstrated in supplemental Fig. 2C.

To confirm that Snail may specifically bind with MMP9 promoter *in vitro*, electrophoresis mobility shift assay (EMSA) was performed using a 25 bp probe, MMP9-proSN, covering the proposed Snail binding motif but not EGR1/SP1 overlapping region (Fig. S2C). As shown in Fig. 4, three EMSA complexes (denoted as SNI, SNII and SNIII) were marginally observed after incubation of MMP9-proSN with nuclear extract of HepG2 treated with TPA for 0 and 1 h. SNI, SNII and SNIII further were increased by 3.0, 5.5 and 6.6-fold, respectively, using the nuclear extract from cells treated with TPA for 2 h, in comparison with that for 1 h. At the 6 h time point, SNI increased by 20%, whereas SNII decreased by 50% compared with that at the 2 h time point. On the other hand, the 2 h-TPA sample was used for competition and antibody blocking analysis. Addition of a 20 bp unlabeled wild type probe (spanning the 5′ region of MMP9-proSN, depicted in Fig. S2C) to the EMSA reaction mix abolished SNI, SNII, and SNIII to basal level, whereas only SNII was significantly reduced if the mutant probe (with 3 bp alteration within the TCACA motif) was added instead. Remarkably, addition of Snail Ab to EMSA reaction mix totally abolished all three complexes to basal level (last lane). Also, addition of EGR1 and SP1 Ab significantly decreased SNII and SNIII by 60–70% but slightly decreased SNI. This result strengthened the possibility that Snail coupled with EGR1 and SP1can specifically bind to the TCACA motif.

### Identification of the TPA-responsive element on ZEB1 promoter

**Figure 5:**
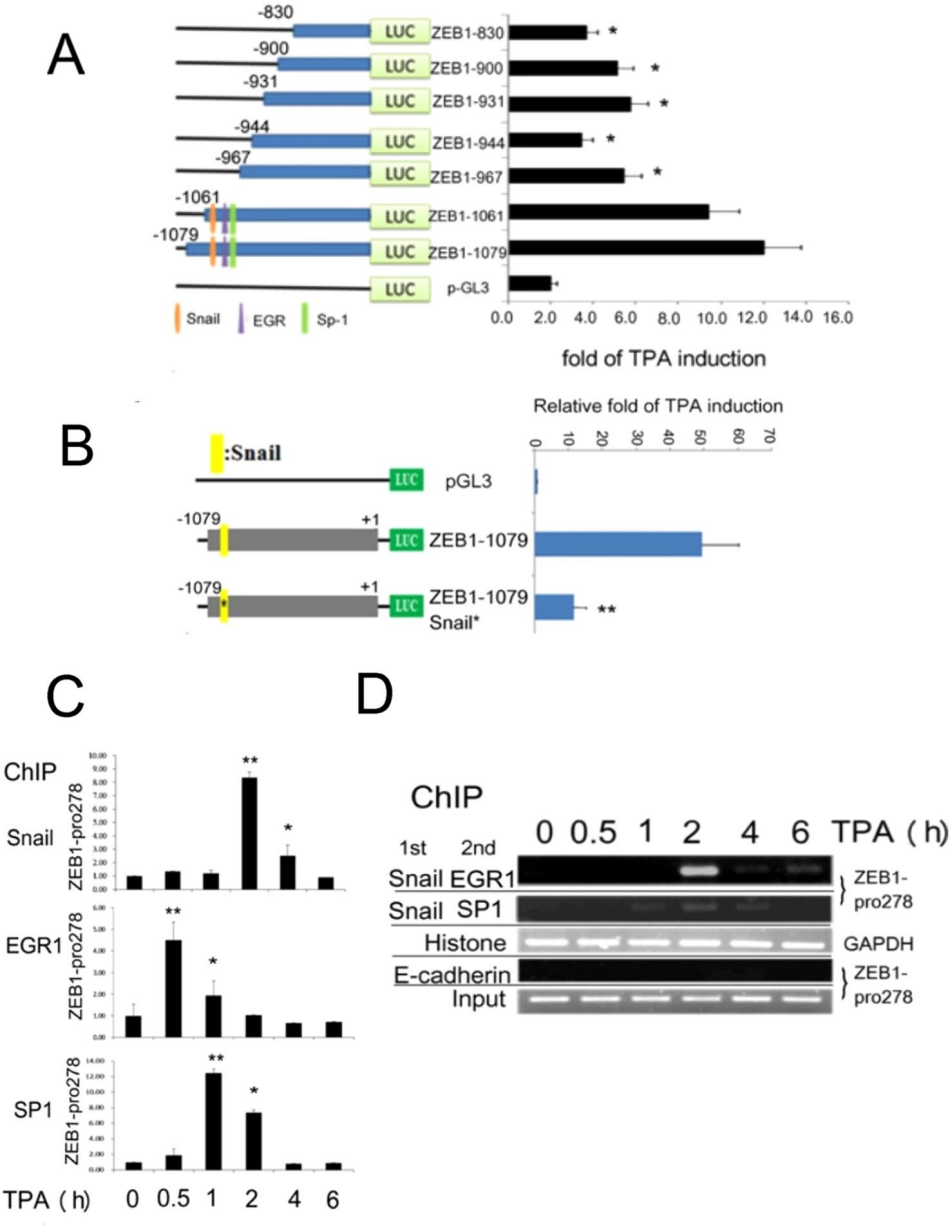
Identification of TPA-responsive region on ZEB1 promoter and TPA induced binding of Snail, EGR and SP1 to specific region of ZEB1 promoter. (**A**) and (**B)**. HepG2 cells were transfected with pGL3 vector, the indicated promoter plasmids with full length promoter (ZEB1–1079), various 5′deleted promoters (**A**) or ZEB1-1079 Snail * with alteration on the proposed Snail binding motif (B) for 24 h. Subsequently, the cells were untreated or treated with 50 nM TPA for 12 h and then dual luciferase assay were performed. The relative TPA-induced promoter activities for each promoter plasmid were quantitated as the activity of TPA-treated *vs*. untreated, taking the data of pGL3 as 1.0 (demonstrated on right panel). (*) represent the statistical significant differences (p < 0.05, N = 3) of relative TPA-induced promoter activity between the indicated samples and the ZEB1-1079 vector group. (**C**) HepG2 cells were treated with 50 nM TPA for the indicated times. ChIP assay for Snail, EGR1 and SP1 binding on ZEB1 promoter region of covering 278 bp within TPA responsive element (ZEB1-pro278) (Fig. S2B). Quantitative PCR were performed for estimating the binding of indicated transcriptional factor on ZEB1-pro278 normalized with the binding of histone3 on GAPDH promoter. Relative binding of each transcriptional factor on ZEB1-pro278 were calculated, taking the data of time zero as 1.0. (**D**) HepG2 cells were treated 50 nM TPA for indicated times. Double ChIP of 1^st^ Snail ChIP followed by 2^nd^ SP-1ChIP or 2^nd^ EGR ChIP was performed. ChIPs of histone binding on GAPDH promoter and E-cadherin on MMP9 promoter are used for positive and negative control, respectively. The figure is representative of two reproducible experiments. The lines between the images in (**D**) are included for dividing gels of different ChIP assays of indicated molecules.

According to the promoter sequence of ZEB1 shown in supplemental Fig. 1B, the proposed Snail binding motif coupled with alternative putative regions of EGR1 and SP1 (EGR1+: 5′gggtgTGGGaggccgaggt3′; SP1+: 5′gaagaGGGCggggagcg3′), were also found in the distal region (searched by Genomatix software). Thus we examined whether they are also required for TPA-induced promoter activation of ZEB1 in HepG2. The full length ZEB1 promoter, ZEB1-1079, was used for constructing various mutants with sequential deletion from 5′ end. Among them, ZEB1-1061 (deletion of 18 bp) contains EGR1 and SP1 overlapping region and upstream proposed Snail motif, whereas the other deletion constructs, ZEB1-967, ZEB1-944, ZEB1-931, ZEB1-900 and ZEB1-830 (deleted by 112, 135, 179 and 249 bp, respectively) doesn’t (Fig. 5A, left panel). As shown in right panel of Fig. 5A, TPA induced similar extent of promoter activation of ZEB1-1079 and ZEB1-1061 by about 5.0~6.0-fold. Remarkably, the TPA-induced promoter activation of ZEB1-967, ZEB1-944, ZEB1-931, ZEB1-900 and ZEB1-830 decreased by 55, 71, 52, 58, and 70%, respectively, as compared with that of ZEB1-1079. This suggested that the TPA-responsive element on ZEB1 promoter locates between −1061 and −830 containing EGR1/SP1 overlapping region and the upstream proposed Snail motif. Moreover, the TPA-induced promoter activation of ZEB1-1079 Snail*, the mutant of ZEB1-1079 with alteration on the proposed Snail binding motif, decreased by 75% as compared with that of the wild type ZEB1-1079 (Fig. 5B). Together, the putative EGR1 and SP1 binding regions coupled with the upstream proposed Snail binding motif were also required for TPA-induced promoter activation of ZEB1.

### TPA triggered binding of Snail, EGR1 and SP1 on ZEB 1 promoter

Further, whether TPA may also trigger binding of Snail, EGR1 and SP1 on ZEB1 promoter was examined in HepG2. ChIP assay was performed using quantitative PCR amplifying −1079~−802 of ZEB1 promoter fragment (ZEB1-pro278) (Fig. S2B) containing the aforementioned Snail, EGR1 and SP1 binding region in ZEB 1 promoter. As shown in Fig. 5C, treatment of the cells with TPA for 2 h dramatically elevated the binding of Snail on ZEB1-pro278 by 8.3-fold, which greatly declined to 2.5-fold at 4 h and return to basal level at 6 h. The TPA-induced binding of EGR1 on ZEB1-pro278 was maximal (4.5-fold) at 30 min, which declined to 1.9-fold at 1 h, whereas that of SP1 was maximal (12.0-fold) at 1 h, partially decreased at 2 h (6.0-fold) and totally declined after 4 h. Thus TPA induced binding of the three transcription factors on ZEB1 promoter containing the sequence motif similar to that within MMP9 promoter.

Whether TPA may trigger co-localization of Snail with EGR1 and SP1 on ZEB1 promoter was further examined by double ChIP assay. As shown in Fig. 5D, treament of HepG2 with TPA for 2 h resulted in a dramatic increase (to about 10-fold) of ZEB1-pro278 amplified from double ChIP of Snail 1^st^/EGR1 2^nd^, which greatly declined at 4 and 6 h. On the other hand, TPA slightly increased ZEB1-pro278 amplified from double ChIP of Snail 1^st^/SP1 2^nd^ at 2 h, indicating weak co-localization between Snail and SP-1. Collectively, these results suggested that TPA can induce the co-localization of Snail with EGR1 on ZEB1 promoter, in similar with that observed on MMP9 promoter.

### Snail is essential for TPA-induced ZEB1/MMP9 promoter activation and gene expression

**Figure 6:**
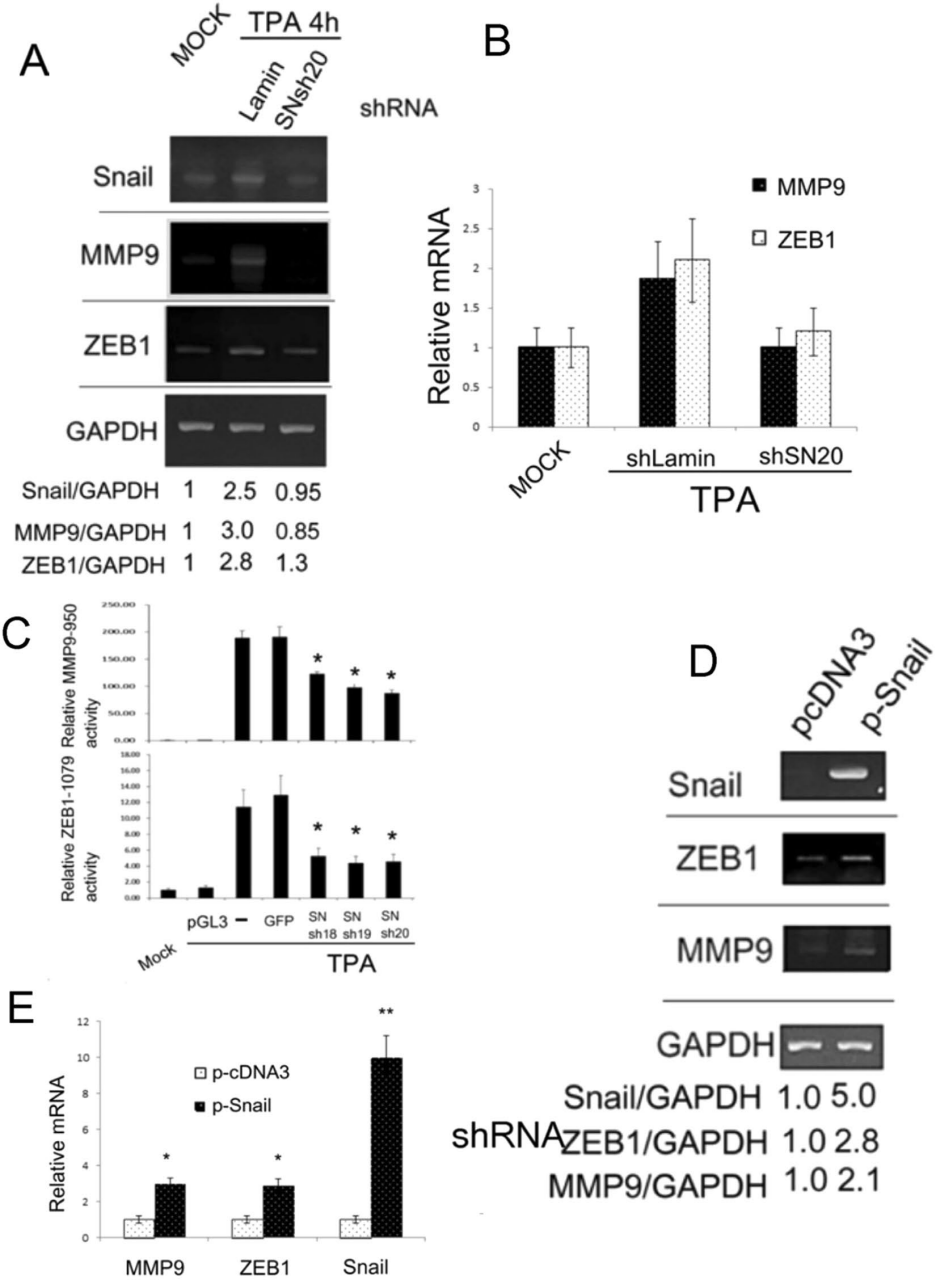
Snail is essential for expression and promoter activation of MMP9 and ZEB1. HepG2 cells were transfected with none (MOCK) or plasmids encoding indicated Snail shRNAs (**A**,**B**,**C**) and control shRNA of Lamin (**A**), (**B**) or GFP (**C**) for 24 h followed by untreated (MOCK) or treated with 50 nM TPA for 4 h (**A**); 4 and 6 h for ZEB1 and MMP9, respectively (**B**); or 12 h (**C**). HCC340 were transfected with pcDNA3 vector or Snail expressing plasmid (p-Snail) for 36 h (**D**) and (**E**). RT-PCR (**A**), (**D**) and quantitative RT-PCR (**B**), (**E**) of Snail, MMP9 and ZEB1 and promoter assay of MMP9-950 (C, upper panel) and ZEB1-1079 (C, lower panel) were performed. In (**A**,**D**), the numbers below each figure are the ratios of relative mRNA based on RT-PCR of indicated transcriptional factor *vs* GAPDH, taking the data of MOCK (**A**) and pcDNA3 (**D**) as 1.0. The results are average of 3 reproducible experiments with C.V. of 7.5%. In (**B**) and (**E**), the relative mRNA was calculated based on real time RT-PCR, taking the data of MOCK (**B**) and pcDNA3 (**E**) as 1.0. In (**C)**, the relative dual luciferase activity of MMP9-950 or ZEB1-1079 was calculated, taking MOCK as 1.0. The data in (**B**), is average of two representative experiment with C.V. of 12%. In (**C**) and (**E**), (**^,^*) represent the statistically significant difference (p < 0.005, N = 3), (p < 0.05, N = 3) between the indicated samples and the control GFP shRNA (**C**) and pcDNA3 (**E**) group.

We further examined whether Snail was required for TPA-induced ZEB1 and MMP9 gene expression, using shRNA technique. Several Snail shRNA expression plasmids, SNsh18, 19, 20, capable of blocking TPA-induced Snail gene expression were employed. The TPA-induced elevation of Snail mRNA at 4 h was abolished by prior transfection of SNsh20, which accompanied the dramatic down regulation of MMP9 and ZEB1 mRNAs by 100 and 82%, respectively, compared with that of control Lamin shRNA in HepG2 (Fig. 6A). Also, in a quantitative real time RT-PCR, ZEB1 and MMP9 mRNA induced by TPA at 4 and 6 h, respectively, were decreased by depletion of Snail mRNA by 80–90% (Fig. 6B). We further examined whether blockade of Snail induction prevented the TPA-induced ZEB1 and MMP9 promoter activation. As shown in Fig. 6C, co-transfection of SNsh 18, 19 or 20 with the promoter plasmid MMP9-950 or ZEB1-1079 suppressed the TPA-induced activation of MMP9-950 (Fig. 6C, upper panel) and ZEB-1079 (Fig. 6C, lower panel) by 40-50%, as compared with that of control GFP shRNA in HepG2.

To further investigate whether elevated expression of Snail *per se* was sufficient for triggering gene expression of ZEB1 and MMP9 mRNA, a Snail expression plasmid, p-Snail, was employed. As shown by RT-PCR in Fig. 6D, transfection of the HCC340 with Snail expression plasmid for 36 h elevated Snail expression to 5.0-fold, accompanied with the increase of ZEB1 and MMP9 mRNA to 2.8 and 2.1- fold, respectively, in HCC340. Quantitative real time RT-PCR also showed a 2.9-fold elevation of ZEB1 and MMP9 mRNA in HCC340 overexpressing Snail (by 10-fold) (Fig. 6E).

## Discussion

### Snail regulates MMP9 and ZEB1 expression through both indirect and direct mechanism

In the past decades, a lot of studies showed that Snail upregulated transcription of MMP9 and ZEB1 in different contexts. In MDCK cell, Snail mediated the TGFβ-induced MMP9 activation by promoting the binding of SP-1/Ets-1 and nuclear factor kappaB (NFkappaB) to the proximal and distal promoter regions, respectively^25^. During oral cancer progression, Snail mediated TGFβ1-induced MMP-9 expression *via* upregulating Ets-1^26^. Moreover, Snail1 activated MMP9 transcription *via* suppression of Cezanne2 in HCC^18^. On the other hand, Snail1 upregulated transcription of ZEB1 by elevating gene expression of Twist and triggering the nuclear translocation of Ets1^27^. Moreover, Snail increased ZEB1 by down-regulation of miR-200^28,29^, known to be negative regulators of ZEB1^29–31^. Collectively, these studies revealed that Snail enhanced gene expression of MMP9 and ZEB1 in an indirect fashion. However, in this study we found Snail, in collaboration with EGR and SP1, can directly activate MMP9 and ZEB1 by binding to a consensus sequence (TCACA), upstream of EGR1 and SP1 binding region. Thus, it appears that both direct and indirect mechanisms are involved in Snail-triggered transcriptional upregulation of ZEB1 and MMP9.

### The consensus Snail binding motif in promoter of Snail-upregulated gene is emerging

It has been established that Snail may bind on E-box to downregulate transcription of a lot of genes including E-cadherin^10,11^. There are two E-box 5′-CACCTG (−972~−988 and −204~−216) on MMP9 promoter, however, according to our deletion mapping analysis, they are not involved in Snail upregulated gene expression in HepG2 (data not shown). In this and our previous study^23^, a novel Snail binding region“TCACA” for upregulating gene expression was identified. Interestingly, a consensus binding site of Drosophila gene snail (sna), 5′-G(or A)A/TG(or A)ACAGGTGC(or T)AC-3′^32^, have been identified in the developing embryo. Lately, this was found to exist in the enhancer elements of the majority of “Snail-activated” genes expressed in the mesoderm at the stages of Snail occupancy^22^. Interestingly, the sequences contained within this binding site (note the sequences indicated as underlined) was similar with the proposed Snail motif “TCACA” identified on the promoters of ZEB1, MMP9 and p15^INK4b^. In the future, whether there are similar consensus binding motifs for Snail to upregulate gene expression among different species is worth investigating.

### The role of Snail-ZEB1/MMP9 transcriptional unit in HCC progression

Previously, the close relationships of Snail with MMP9 and ZEB1 were frequently observed in the molecular pathway triggering HCC progression. For example, overexpression of ZEB1 and Snail were simultaneously induced by14-3-3ε, required for EMT and migration of HCC^17^. Moreover, overexpression of Tetraspanin CD151 in HCC facilitated MMP9 expression through a Snail mediated pathway^33^. In addition, Axl/14-3-3ζ signaling caused up-regulation of tumor-progressive TGF-β target genes including MMP9 and Snail^34^. Consistently, we found Snail was required for TPA-induced cell migration^35^ and transcriptional activation of MMP9 and ZEB1 (Fig. 6A and B) in HepG2. Moreover, overexpression of Snail triggers gene expression of ZEB1 and MMP9 (Fig. 6D and E). Thus, the Snail-ZEB1/MMP9 transcriptional unit may serve as molecular machinery for triggering HCC progression.

## Conclusion and Perspective

In this study, we establish a general transcriptional mechanism by which Snail upregulates gene expressions. Specifically, Snail directly binds to a consensus motif in the distal promoter upstream of the EGR/SP-1 binding region. By Genomatix software, we found the similar sequence architectures were also revealed in promoters of several other Snail-upregulated mesenchymal genes such as fibronectin^36^ and lymphoid enhancer-binding factor (LEF)^37^ (Fig. S1C), which are also involved in HCC progression^38,39^. In a preliminary time course analysis, we found fibronectin can be induced by TPA at 2 h and LEF can be biphasically induced by TPA by 3.2 and 3.5-fold, at 1 and 4 h, respectively, in HepG2 (Fig. S5). In the future, whether these genes are also upregulated by Snail in a similar fashion as MMP9 and ZEB1 is worth investigating.

## Materials and Methods

### Cell culture, chemical

The cultured conditions for HCC340 and HepG2 cells were the same as described in our previous reports^24,35^. HCC 340 is a patient-derived hepatocellular carcinoma cell line from Buddhist Tzu Chi Hospital, Taiwan^24^. Tetradecanoyl phorbol acetate (TPA) was purchased from Sigma-Aldrich (Poole, UK). The snail expression plasmid, p-Snail, driven by CMV promoter within the pcDNA3 vector, is a gift from Dr. Cheng K.K. in Tzu Chi university.

### Constructions of various promoter plasmids for deletion mapping

The promoter regions in the full length promoter plasmids MMP9-950 and ZEB1-1079 were amplified from genomic sequence of MMP9 and ZEB1 encompassing 950 bp and 1079 bp, respectively, upstream of translation start site. The PCR products were ligated into pGL3 vector (Promega, Madison, WI, USA). The promoter plasmids MMP9-870, MMP9-832, MMP9-812, MMP9-615, MMP9-341 and ZEB1-1061, ZEB1-967, ZEB1-830 were derived from MMP9-950 and ZEB1-1079, respectively, by double digestion with various restriction enzymes followed by filling in the restriction site overhangs by Klenow enzyme. Subsequently, the digested DNA fragments were ligated into pGL3 vector.

### Site-directed mutagenesis on MMP9 and ZEB1 promoters

The full length promoter plasmids MMP9-950 and ZEB1-1079 were used as templates for site-directed mutagenesis using a GeneEditor *in vitro* site-directed mutagenesis system (Promega, Madison, WI, USA) to obtain various mutant promoters according to the manufacturer’s protocol. MMP9-950 Snail* and MMP9-950 E/S* are mutant promoters with alteration of 3 bp in proposed Snail binding motif (TCACA) and the EGR1 overlapping (CCCACC), respectively, on MMP9-950, whereas MMP9-950 PAX6* and MMP9-950 PLZF* are mutants with alteration on the putative binding motifs of PAX6 and PLZF, respectively. ZEB1-1079 snail* is the mutant of ZEB1-1079 with alteration on Snail binding motif (TCACA). The bases changed in the site-directed mutagenesis for proposed Snail binding motif (TCACA) and the EGR1/SP1 overlapping region were the same as those described in our previous report^23^.

### Dual and single luciferase promoter assay

Luciferase reporter gene assays for activities of various promoter plasmids were conducted using the Dual-Luciferase assay system (Promega, Madison, WI, USA). The activity expressed by *Renilla* luciferase vector was used as an internal control. The promoter activities were normalized as the activity of experimental reporter to that of the internal control for minimizing experimental variability. The single Luciferase assay (Promega, Madison, WI, USA) in Fig. S3 was performed without addition of the *Renilla* luciferase vector as internal control.

### Chromatin immunoprecipitation (ChIP) assay

ChIP assay was performed using a modification of the standard protocol. Briefly, chromatins were cross-linked with transcription factors or other chromatin-associated proteins, sheared by sonication into fragments (100–1000 bp) and immunoprecipitated using polyclonal antibodies against Snail, EGR-1 and SP-1. As a positive control, antibody of histone H3 (Santa Cruz Biotechnology, California, USA) was used to precipitate the histone-bound GAPDH promoter. The recovered DNA was analyzed by PCR with primers flanking the putative transcription factor binding sites as indicated. For quantitation, the gels were scanned and the intensity of each PCR band was estimated with gel digitizing software, un-scan-it gel v. 5.1. The primers used for PCR of the ChIP fragments MMP9-pro179, MMP9-pro155, ZEB1–pro278 and GAPDH promoter were designed using primer 3 software as shown in Table 1. The PCR program was 95 °C for 10 minutes followed by 45 cycles of 95 °C for 50 seconds, 60 °C for 40 seconds and 72 °C for 1 minute.

**Table 1:** Primers used for ChIP assays.

Gene	Primer sequence	Product size
GADPH	F: 5′ TACTA GCGGT TTTAC GGGCG3′	166 bp
	R: 5′TCGAACAGGAGGAGCAGAGA GCGA 3′	
ZEB1-pro278	F: 5′AATCAGAATCTATCAGGTTCA3′	278 bp
	R: 5′TTAGTAGAGCGGAATGAGTAA3′	
MMP9-pro179	F: 5′CAACCTACAGTGTTCTAAACA3′	179 bp
	R: 5′TAGAAAACAGCAGACATGGTTTA3′	
MMP9-pro165	F: 5′ TCCTC ACATC AATTT AGGGA3′	165 bp
	R: 5′AGGGC AGTAA AGGGG ACAGT 3′	
MMP9-pro155	F: 5′CGATT AGGAA TGAGC CACCA 3′	155 bp
	R: 5′TCCCT AAATT GATGT GAGGA TT 3′	

### RT-PCR

For analyzing gene expression of Snail, MMP9 and ZEB1, total mRNA was isolated by Trizol reagent (Thermo Scitific, Dharmacon, US). After reverse transcription, each of the cDNA was amplified by PCR. The PCR program was 95 °C for 10 minutes followed by 25–30 cycles of 95 °C for denaturing (50 seconds), 60 °Cfor annealing (40 seconds) and 72 °C for extension (40 seconds). For quantitation, the gels were scanned and the intensity of each PCR band was estimated with gel digitizing software un-scan-it gel v. 5.1. The primers used for PCR of the indicated genes were shown in Table 2.

**Table 2:** Primers used for RT-PCR and real time PCR in gene expression analysis.

Gene	Primer sequence	Product size
GADPH	F: 5′ACC ACA GTC CAT GCC ATC AC 3′	450 bp
	R: 5′TCC ACC ACC CTG TTG CTG TA3′	
Snail	F: 5′AAG CTT CCA TGG CGC GCT CTT TCC TCG TCA GGA AGC CC3′	795 bp
	R: 5′GGA TCC TCA GCG GGG ACA TCC TGA GCA GCC GGA CTC TTG3′	
ZEB1	F: 5′TTC AGC ATC ACC AGG CAG TC3′	736 bp
	R: 5′GAG TGG AGG AGG CTG AGT AG3′	
MMP9	F: 5′TGT ACC CTA TGT ACC GCT TCA CT3′	489 bp
	R: 5′AGA AGA AAA GCT TCT TGG AGA GC A3′	
Fibronectin	F: 5′AAGGAG AAG ACCGGA CCA AT	314 bp
	R:GGC TTG ATG GTT CTC TGG AT	
LEF1	F:TGGCA GCCCT ATTTC AGTTT	280 bp
	R:CAAAG GCTGT GCTTG CTTTT	

### Quantitative RT-PCR

Real-time PCR for single and double ChIP, and gene expression of Snail, MMP9, and ZEB were performed by QuantiTect SYBR PCR kit (Qiagen, Crawley, UK) using ABI 7300 real-time PCR system (Applied Biosystems, Foster City, CA, USA). The primer sequences used for MMP9-pro165 were shown in Table 1. GAPDH was included as an internal control. HotStar Taq DNA polymerase was used for primer extension. PCR mixtures were pre-incubated at 95 °C for 15 min to activate the polymerase. Each of the 40 PCR cycles consisted of 16 s of denaturation at 94 °C, annealing of primers for 30 s at 55 °C and 15 s of extension at 72 °C. The relative amounts of ChIP PCR product were calculated using 7300 system sds Software. In Real-time PCR for quantitating mRNA of Snail, MMP9, ZEB, fibronectin and LEF, each of the 40 PCR cycles consisted of 15 s of denaturation at 95 °C, annealing of primers for 30 s at 60 °C and 15 s of extension at 72 °C. The primer sequences used were shown in Table 2. GAPDH was included as internal control.

### Electrophoresis mobility shift assay (EMSA)

Probes for EMSA were prepared as biotin-labeled DNA promoter fragments using Biotin 3′ End DNA Labeling Kit (Pierce) according to manufacturer’s procedure. The sequence of the biotin-labeled probe (25 bp) and un-labeled competitors of MMP9 promoter (20 bp) are 5′TTTAATCC***TCACAT***CAATTTAGGGA3′ and TTTAATCC***TCACA***TCAATTT (wild type)/TTTAATCC***TAGAA***TCAATTT (mutant), respectively. For EMSA reactions, 15 μg of nuclear extracts were incubated with 1 μg/μl polydI. dC, and 1 μg/μl of biotin-labeled DNA probe. For competition assays, 200-fold of unlabeled DNA probes was added in reaction mix. For antibody blocking, 15 μg of nuclear extracts were pre-incubated with 2 μg of various antibodies (Santa Cruz Biotechnology, California, USA) and 1 μg/μl of biotin-labeled DNA probe at room temperature for 30 minutes. After separation by 6% polyacrlamide gel, the DNA-protein complexes were detected by avidin-linked HRP using Chemiluminescent Nucleic Acid Detection Module (Pierce).

### Western blot

Western blot was performed as described in our previous report^35^. The antibodies against Snail, EGR1, SP1, MMP9 and ZEB1 were from Santa Cruz Biotechnology (California, USA). For quantitation, the intensity of each specific band was estimated with gel digitizing software un-scan-it gel v. 5.1.

### shRNA technology

Lentiviral plasmids each encoding shRNA targeting different regions of the indicated mRNA were obtained from RNAi Core Laboratory (Academia Sinica, Taiwan). Cells at 60% confluence were transfected with shRNA, using lipofectamin transfection reagent (Invitrogen Ltd, Renfrew, UK) according to the manufacturer’s protocol. Lentiviral plasmid encoding human Lamin A shRNA was used as a control shRNA. The sequence of shRNA fragments targeting different regions of Snail were the same as those used in our previous study^23^.

### Zymography

Briefly, HepG2 was treated with TPA for appropriate times, then the conditioned mediums were collected and subjected to 0.1% gelatin-8% SDS-PAGE electrophoresis. Subsequently, gels were washed with 2.5% Triton X-100 and incubated in developing buffer (0.5 M Tris-HCl, PH 7.8, 2 M NaCl, 0.05 M CaCl_2_, and 0.2% Brij 35) overnight at 37 °C followed by staining with Coomassie Brilliant Blue G250 (Beyotime, China) for 2 h. After that, the gels were washed with destaining solution (5% methanol and 10% acetic acid in ddH_2_O).

### Statistical analysis

Data were analyzed using Student’s t-test in Excel. All the quantitative studies were performed at least in triplicate, as appropriate. Statistical significance between groups was indicated by *P < 0.05 and **P < 0.005.

## Electronic supplementary material

combined supplemental figures
