# Author Correction: Snail collaborates with EGR-1 and SP-1 to directly activate transcription of MMP 9 and ZEB1

**DOI:** 10.1038/s41598-018-28913-w

**Published:** 2018-09-18

**Authors:** Wen-Sheng Wu, Ren-In You, Chuan-Chu Cheng, Ming-Che Lee, Teng-Yi Lin, Chi-Tan Hu

**Affiliations:** 1 Institute of medical biotechnology, college of Medicine, Tzu Chi University, Hualein, Taiwan; 2 Department of Surgery, Buddhist Tzu Chi General Hospital, School of Medicine, Tzu Chi University, Hualien, Taiwan; 3 Department of Laboratory Medicine, Hualien Tzu Chi Hospital, Buddhist Tzu Chi Medical Foundation, Hualien, Taiwan; 4 Research Centre for Hepatology, Department of Internal Medicine, Buddhist Tzu Chi General Hospital and Tzu Chi University, Hualien, Taiwan

Correction to: *Scientific Reports* 10.1038/s41598-017-18101-7, published online 19 December 2017

This Article contains errors in Figures 6b and 6c where the ‘shRNA’ axes labels are missing. Additionally, in Figure 6D, ‘shRNA’ is erroneously included as part of the ‘ZEB1/GAPDH’ label. The correct Figure 6 appears below as Figure 1.

**Figure 1:**
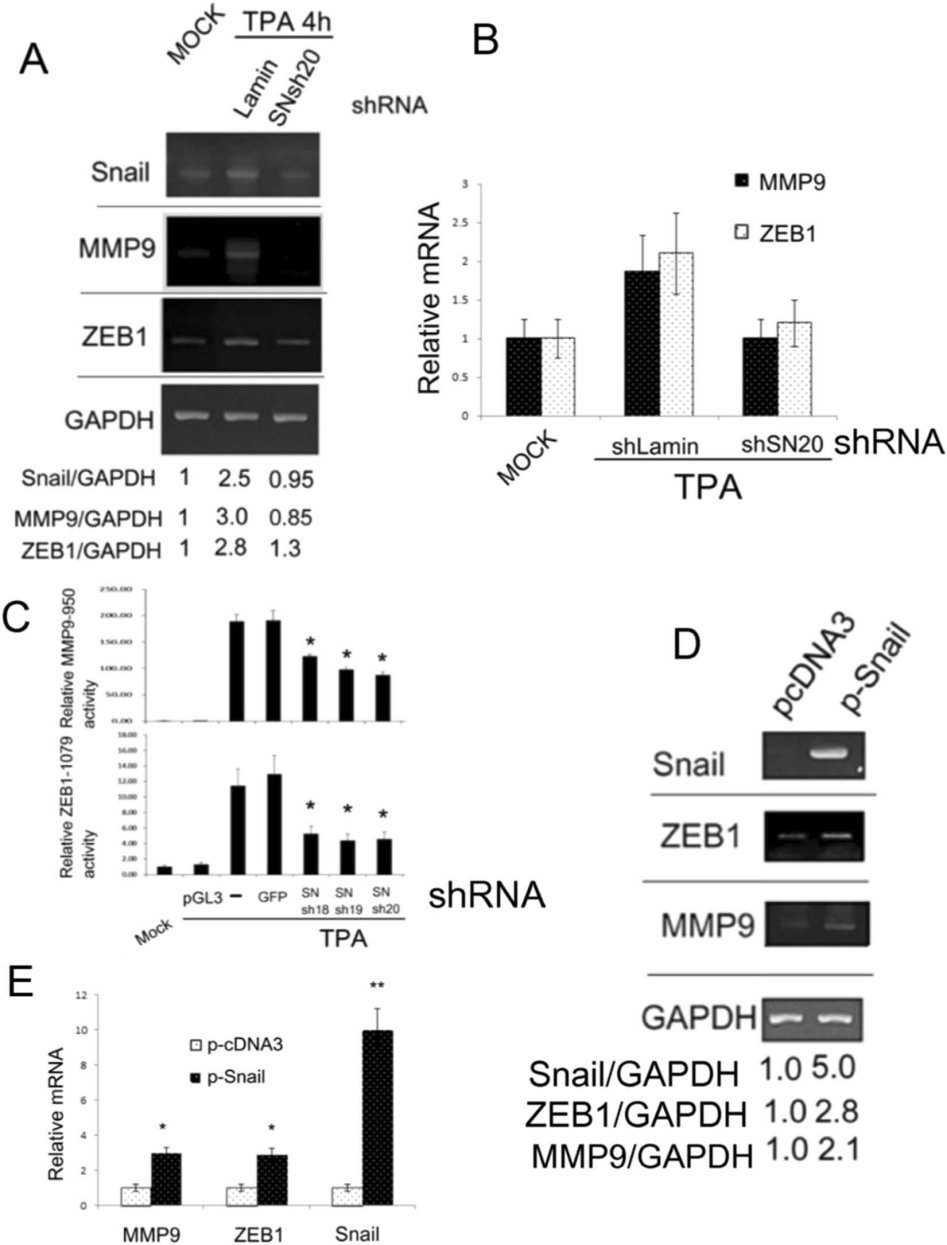
Snail is essential for expression and promoter activation of MMP9 and ZEB1. HepG2 cells were transfected with none (MOCK) or plasmids encoding indicated Snail shRNAs (**A**,**B**,**C**) and control shRNA of Lamin (**A**), (**B**) or GFP (**C**) for 24 h followed by untreated (MOCK) or treated with 50 nM TPA for 4 h (**A**); 4 and 6 h for ZEB1 and MMP9, respectively (**B**); or 12 h (**C**). HCC340 were transfected with pcDNA3 vector or Snail expressing plasmid (p-Snail) for 36 h (**D**) and (**E**). RT-PCR (**A**), (**D**) and quantitative RT-PCR (**B**), (**E**) of Snail, MMP9 and ZEB1 and promoter assay of MMP9-950 (C, upper panel) and ZEB1-1079 (C, lower panel) were performed. In (**A**,**D**), the numbers below each figure are the ratios of relative mRNA based on RT-PCR of indicated transcriptional factor *vs* GAPDH, taking the data of MOCK (**A**) and pcDNA3 (**D**) as 1.0. The results are average of 3 reproducible experiments with C.V. of 7.5%. In (**B**) and (**E**), the relative mRNA was calculated based on real time RT-PCR, taking the data of MOCK (**B**) and pcDNA3 (**E**) as 1.0. In (**C)**, the relative dual luciferase activity of MMP9-950 or ZEB1-1079 was calculated, taking MOCK as 1.0. The data in (**B**), is average of two representative experiment with C.V. of 12%. In (**C**) and (**E**), (**^,^*) represent the statistically significant difference (p < 0.005, N = 3), (p < 0.05, N = 3) between the indicated samples and the control GFP shRNA (**C**) and pcDNA3 (**E**) group.

