# Supplementary material for: Snail collaborates with EGR-1 and SP-1 to directly activate transcription of MMP 9 and ZEB1
Source: Sci Rep. 2017 Dec 19;7:17753. doi: 10.1038/s41598-017-18101-7 (PMC5736704; doi:10.1038/s41598-017-18101-7)
Supplement: Supplementary file 1 — combined supplemental figures [file 41598_2017_18101_MOESM1_ESM.pdf]

Snail collaborates with EGR-1 and SP-1 to directly activate transcription of MMP 9 and ZEB1

Wen-Sheng Wu*, Ren-In You*, Chuan-Chu Cheng*, Ming-Che Lee[&], Teng-Yi Lin[$], Chi-Tan Hu[#1]

[#]Research Centre for Hepatology, Department of Internal Medicine, Buddhist Tzu Chi General Hospital and Tzu Chi University, Hualien, Taiwan. *Institute of medical biotechnology, college of Medicine, Tzu Chi University, Hualein, Taiwan. [&]Department of Surgery, Buddhist Tzu Chi General Hospital, School of Medicine, Tzu Chi University, Hualien, Taiwan. [$]Department of Laboratory Medicine, Hualien Tzu Chi Hospital, Buddhist Tzu Chi Medical Foundation, Hualien, Taiwan

[1]Correspondence: Chi-Tan Hu. Division of Gastroenterology, Department of Internal Medicine, Buddhist Tzu Chi General Hospital, No. 707, Chung Yang Rd, Sec. 3, Hualien 970, Taiwan. e-mail: **chitan.hu@msa.hinet.net**；Phone: +886-3-8561825 ext 3224；Fax: +886-3-8567043.

Supplemental Fig. 1 promoter region of MMP9, ZEB1, fibronectin and lymphoid enhancer-binding factor 1 (LEF1) exhibit similar TCACA and down stream EGR1/SP1 binding region

(A) Homo sapiens matrix metallopeptidase 9 (gelatinase B, 92kDa gelatinase, 92kDa type IV collagenase) (MMP9), RefSeqGene on chromosome 20, NCBI Reference Sequence: NG_011468.1

-911

GCCTTAGTAATTAAAACCAATCACCACCATCCGTTGCGGACTTACAACCTACAGTGTTCTAAAC#ATTTTATATGTTT
GATCTCATTTAATCCTCACATCAATTTAGGGACA*AAGAGCCCCCCACCCCCCGTTTTTTTTTTTACAGCTGAGGAAACACTTC
AAAGTGGTAAGACATTTGCCCGAGGTCCTGAAGGAAGAGAGTAAAGCCATGTCTGCTGTTTTCTAGAGGCTGCTACTGTCCCCT
TTACTGCCCTGAAGATTCAGCCTGCGGAAGACAGGGGGTTGCCCCAGTGGAATTCCCCAGCCTTGCCTAGCAGAGCCCATTCCT
TCCGCCCCCAGATGAAGCAGGGAGAGGAAGCTGAGTCAAAGAAGGCTGTCAGGGAGGGAAAAAGAGGACAGAGCCTGGAGT
GTGGGGAGGGGTTTGGGGAGGATATCTGACCTGGGAGGGGGTGTTGCAAAAGGCCAAGGATGGGCCAGGGGGATCATTAGTT
TCAGAAAGAAGTCTCAGGGAGTCTTCCATCACTTTCCCTTGGCTGACCACTGGAGGCTTTCAGACCAAGGGATGGGGGATCCC
TCCAGCTTCATCCCCCTCCCTCCCTTTCATACAGTTCCCACAAGCTCTGCAGTTTGCAAAACCCTACCCCTCCCCTGAGGGCCTG
CGGTTTCCTGCGGGTCTGGGGTCTTGCCTGACTTGGCAGTGGAGACTGCGGGCAGTGGAGAGAGGAGGAGGTGGTGTAAGCC
CTTTCTCATGCTGGTGCTGCCACACACACACACACACACACACACACACACACACACACACACACACCCTGACCCCTGAGTCA
GCACTTGCCTGTCAAGGAGGGGTGGGGTCACAGGAGCGCCTCCTTAAAGCCCCCACAACAGCAGCTGCAGTCAGACACCTCT
GCCCTCACC**ATG**

#: TPA-responsive element (-832 ~ -771) is marked with

* Searching results of EGR1 and SP1 binding regions downstream of TCACA by Genomatix software

EGR1(-) : 5’ cgggGGGTggggggctctt 3’

EGR1(+): 5’aagagccccccACCCcccg 3’

SP1(-): 5’ acgggGGGTgggggct 3’

SP1(+): 5’agccccCCACcccccgt3’

EGR/SP1 (+) overlapping region: agccccccACCCcccg

Transcription Factor Binding Sites common to at least 100% of the sequences

Graphical View | Match Summary Table | Common Matches

Common Match Table (click on headers to sort columns)

#	Seq. name	Gene Id	Symbol	Family	Matrix	Start	End	Strand	Matrix sim.	Core sim.	Sequence
1	TempSeq_H2ScGWr5			V$EGRF	V$NGFIC.01	1	19	-	0.814	0.755	cgggGGGTggggggctct

Common Match Table (click on headers to sort columns)

#	Seq. name	Gene Id	Symbol	Family	Matrix	Start	End	Strand	Matrix sim.	Core sim.	Sequence
9	TempSeq_H2ScGWr5			V$SP1F	V$SP1.01	4	20	-	0.896	0.772	acgggGGGTggggggct

(B) Homo sapiens zinc finger E-box binding homeobox 1 (ZEB1), RefSeqGene on chromosome 10

NCBI Reference Sequence: NG_017048.1

-1132

TTGTGGTTATCTGTATGGTCTTTTCAGAAATCCCAAAACTTGTACCAAGTCAAGGATAAAATAAGATAAAA#TCAG
CAATCTATCAGGTTCAGAGATCACATCTGTCAGCCGATGCTTCTTGCCTTAAGGTCCTGCACGGCGATGACCGCTCATTTA
GGAAGGAATTCATGGCCTGTGGATACCTTAGCTCTGAGTCCTGCCACCTAGGATCCCACGGTTCTACGCGAG*GAAGAGGGCG
GGGAGCGCGGACC*GGGTGTGGGAGGCCGAGGTGACAGCAGGTGAGGGCCGGGTGCGGATGGGGAAGTGAGACAAGCACC
GTGTGGGTATTACTCATTCCGCTCTACTAAGGAGGCTGCTGGCAAGCGGAACTTCTAGCCTCTCTTTCAATCCAGCTGAAGTTC
AATCTCATTGAAGTCACTTCCCATCCCGGTTCGCTTGGGGGGAAACCAGGCGTCCCTGGAAGGGAAGGGAAGGGAGTCCGGG
CTGCGCGGGTCAGGTAGCCTCTCTCCGGTCGCCGCGTGTCCTCGCCGTCCCCAAACCTGCCCTTCCCCTCATCAAGGGAACTC
CCCGGGGAGTCCAGACCGCGATCCCTTTCCTTGGCCCCGGGGTGCGGGGGGCGGACACGCGAGGCGTGGGACTGATGGTAGC
CCTGCCTCCAGGAAGCAGGCAGGCGGGGACCTCTGGGCGCTCGGAGGGGCAGCTCCGAGGGCACAGGGTACAGGGAGAATC
AGCCAGATCCCTCCCTGCCCCGGGCAGCCGCGGCGGGTGTGGCCAGCGCGGAGGCAGGACGCCGCCGAGCCTCCAACTTTAC
CTTTCCAACTCCGACAGCCCGTCGCCTTTCTGACCGCGTCCCTACGGTTTCCCGGCATCCGCCTCCCTCTCCCCACCACACCTG
AGGAAAACTTTTCCCTCGCCCCTCAATTCAAATTCAGCAGTGCCCACGGTTGCCGCAAACCGCCCGGTCCCTAGCAACAAGGT
TCCGGCCGTAGAGCGAGAGCCTCTAGGTGTAAGGAAGGTGATGTCGTAAAGCCGGGAGTGTCGTAAACCAGGTGCGGTGGGG
AGGGGGGAGGGGTGGAGGCGGAGGGGTGGGGGGAAGGGGGAGGGAGGGGGAGGGGTGACTCGAGCATTTAGACACAAG
CGAGAGGATC**ATG**GCGGATGGCCCCAGGTGTAAGCGCAGAAAGCAGGCGAACCCGCGGCGCAATAACGGTGAGTGGCGGAG
GGGACCGGGGAGCGGCGGAGTCAGGGGGAGCTGGGCAGCCGGGGCGCCCCCGGGGGTGAGGGGGGCGAGCCGGGCTGGGG
GCAGCCGGGGCAGGGACGGCAAAGTGGAGTGGGAAAGTAGAA

#: TPA-responsive element (-1061~-830) is marked with

* Searching results of EGR1 and SP1 binding regions downstream of TCACA by Genomatix software

EGR-1 (+) 5’ gggtgTGGGaggccgaggt 3’

Sp1(+): 5’ gaagaGGGCggggagcg 3’

EGR

Common Match Table (click on headers to sort columns)

#	Seq. name	Gene Id	Symbol	Family	Matrix	Start	End	Strand	Matrix sim.	Core sim.	Sequence
43	TempSeq_8mUOFsy9			V$EGRF	V$WT1.01	152	170	+	0.922	1.000	gggtgTGGGaggccgag[illegible]

SP1

Common Match Table (click on headers to sort columns)

#	Seq. name	Gene Id	Symbol	Family	Matrix	Start	End	Strand	Matrix sim.	Core sim.	Sequence
27	TempSeq_8mUOFsy9			V$SP1F	V$SP1.03	129	145	+	0.958	1.000	gaagaGGGCggggagcg[illegible]

(C) **Homo sapiens fibronectin 1 (FN1), RefSeqGene on chromosome 2**

>gi|238018066|ref|NG_012196.1| Homo sapiens fibronectin 1 (FN1), RefSeqGene on chromosome 2

-683

CTCAAACACTACCACCACCCCCAATAAAAAAGAAAAGGGAAGGGGGAGCGTCTTGCAACCCCTTCGCTTCACACAAGTCC

AGCCACTCCCTTTCCTCCCAGCCGCTTCCCATCCCTTCCCCCATCCCCTAAAAAGTTTGATGACCGCAAAGGAAACCGAAAA

AAAGTTGTCTTGCCCCAGTCCTGGCGGGCCATCAGCATCTCTTTTGTTCGCTGCGAACCCACAGTCCCCCGTGACGTCACCC

GGAGCCCGGGCCAATCGGCGCGCGGTCGGCTGCGGCGG*CCGGCGGGCGGGCGGGCGGGTGGGGTGGGGCGGGGCGGGG

ACAGCCCGGCGGGTCTCTCCTCCCCCGCGCCCCGGGCCTCCAGAGGGGCGGGAGGGGACCGTCCCATATAAGCCCCGGCTC

CCGGCGCTCGGACGCCCGCGCCGGCTGTGCTGCACAGGGGGAGGAGAGGGAACCCCAGGCGCGAGCGGGAAGAGGGGAC

CTGCAGCCACAACTTCTCTGGTCCTCTGCATCCCTTCTGTCCCTCCACCCGTCCCCTTCCCCACCCTCTGGCCCCCACCTTCT

TGGAGGCGACAACCCCCGGGAGGCATTAGAAGGGATTTTTCCCGCAGGTTGCGAAGGGAAGCAAACTTGGTGGCAACTTG

CCTCCCGGTGCGGGCGTCTCTCCCCCACCGTCTCAACATGCTTAGGGGTCCGGGGCCCGGGCTGCTGCTGCTGGCCGTCCA

GTGCCTGGGGACAGCGGTGCCCTCCACGGGAGCCTCGAAGAGCAAGAGGCAGGCTCAGCAAATGGTTCAGCCCCAGTCCC

CGGTGGCTGTCAGTCAAAGCAAGCGTGAGTACTGACCGCGGGCTGAAACAGGCTGCCTCAGGGATGGGACCCTAAAGCCG

ACCAAAGTTGGGGCTGAAGTTTTGTGCGCGCGCGTGTGTGCGAGTGTGTGCGCGCTTTACTGAGAGAAACCAGCTGTGCAC

ACAAAAGGACCGAGTTTTGAGCACGCTGGTTCTGAGGGCCTGGGATGATAAGACCGTGCATTGGAGGACGAGGACTCTGCG

ACTTTCCCGTGTTCTAATAAATTCTGCACGTTCAGATTGTCCTTCTAGGAATTAACCAAAACTTGCCTTTAAAGAGAAAAATG

ATGCATGTCTATAAATTTTCCGTCTGGGATTAGTGTGGTCCTTACTGCTACTTATTTCCTTCTGTTAAATAATTGGTCAAATATTT

TCAACATGGGGGTGGAAAGGGGGTATTGAAATAGCTGTCTTGTTTCTAACTAACTTGGAAGAGATGTAATTGGTTCAGACCT

CTTTAGGGCCGCTCAGGATACTTCACCAAGAACAGAGGTTGGAATTCTTTCCGTTTTTCAAAGACACACCCTCCTTTTGCTTT

GAGAAAGCTGCTTAAAGTTGTCCTTTTTGACTATTACTCCAAAAGAATATTTAAGTTCCTTGCATGTTTTAAAAATGTGACTTC

AATTGTCTGCCTTCCAAAATGTTTCCAACTTTTTTATGTAGACCCCTGGCCAGATGGAAATGACATCATTGTATATAACTTTTA

GCAAAGTTAAAAGGAAAAAAATATGTACGTCAATATTCACATGAAGAAAATTCCATAATTTTGGGAAAAGGAGAAATGCAAA

TGTAACGTTTTCCTTCAATTATTTGCAGCCGGTTGTTATGACAATGGAAAACACTATCAGATAAATCAACAGTGGGAGCGGAC

CTACCTA

* Searching results of EGR1 and SP1 binding regions downstream of TCACA by Genomatix software

EGR-1 (-): 5’ cggcgggcgGGCGggcggg 3’

Sp1 (+): 5’ ccggcGGGCgggcgggc 3’

EGR/SP1 overlapping region in FN1: cggcGGGCgggcgggc

EGR1

Common Match Table (click on headers to sort columns)

#	Seq. name	Gene Id	Symbol	Family	Matrix	Start	End	Strand	Matrix sim.	Core sim.	Sequence
140	TempSeq_DKm4zj4E			V$EGRF	V$EGR2.02	259	277	-	0.943	0.850	gcgGGGGaggagagacccg
77	TempSeq_DKm4zj4E			V$EGRF	V$EGR1.04	211	229	+	0.888	1.000	cggcgggcgGGCGggcggg

SP1

#	Seq. name	Gene Id	Symbol	Family	Matrix	Start	End	Strand	Matrix sim.	Core sim.	Sequence
160	TempSeq_DKm4zj4E			V$SP1F	V$SP1.01	290	306	+	0.963	1.000	cagagGGGCgggagggg
76	TempSeq_DKm4zj4E			V$SP1F	V$SP1.03	210	226	+	0.936	1.000	ccggcGGGCgggcgggc

Common Match Table (click on headers to sort columns)

(D) Homo sapiens lymphoid enhancer-binding factor 1 (LEF1), RefSeqGene on chromosome 4

-789

TATAAGCAGCGCCCGGTCCTTCCTTCTCTCGCCAAGTTGCCTGATCCTTCCCTCCAGGCGCGCGCACACACCACACTCA
CACACCCCAAAACCAAGACTCGTCCTACAGGATCTGGAAAAGAAAAAGAAAAAAAAGCCCTCAATCACCACCTCCTTCT
*CGCCGACTCCCCCTCACCCCCCGCCTCCCCTCCAGCGGGCAGCCAAGGAGAGCTAGAGGCGGGGGAGGGGAGAGGGAG
GAGAAGCGACGCAAGTGGGTAGCTTTTCAGCGCCGGCGAGGCGCGGGAGGAGGAGAAGCAGTGGGGAGGCGCAGCCGC
TCACCTGCGGGGCAGGGCGCGGAGGAGGGACCCGGGCTGCGCGCTCTCGGGCCGAGGAACCAGGACGCGCCCGGAGCCT
CGCACGCGGCCAAGCTCGGGGCGTCCCCTCCCCTCGGCCGGGCGAACTCAAGGGGCGCAGCTCTTTGCTTTGACAGAGCT
GGCCGGCGGAGGCGTGCAGAGCGGCGAGCCGGCGAGCCAGGCTGAGAAACTCGAGCCGGGAACAAAGAGGGGTCGGAC
TGAGTGTGTGTGTCGGCTCGAGCTCCGGGCAGAGGCATTTGGGCCCGAGGCCCCCGCTGTGACTCCCCGAGACTCCGCAG
TGCCCTCCACTGCGGAGTCCCCGCGCTTGCCGGCAAAAACTTTATTCTTGGCAAACTTCTCTTTCTCTTCCCCTCCTCCTCG
GCCCCCATCTTCTGCTCCTCCTCCTTCTCTAGCAGATTAAATGAGCCTCGAGAAGAAAAACCGAAGCGAAAGGGAAGAAA
ATAAGAAGATCTAAAACGGACATCTCCAGCGTGGGTGGCTCCTTTTTCTTTTTCTTTTTTTCCCACCCTTCAGGAAGTGGAC
GTTTCGTTATCTTCTGATCCTTGCACCTTCTTTTGGGGCAAACGGGGCCCTTCTGCCCAGATCCCCTCTCTTTTCTCGGAAAA
CAAACTACTAAGTCGGCATCCGGGGTAACTACAGTGGAGAGGGTTTCCGCGGAGACGCGCCGCCGGACCCTCCTCTGCAC
TTTGGGGAGGCGTGCTCCCTCCAGAACCGGCGTTCTCCGCGCGCAAATCCCGGCGACGCGGGGTCGCGGGGTGGCCGCCG
GGGCAGCCTCGTCTAGCGCGCGCCGCGCAGACGCCCCCGGAGTCGCCAGCTACCGCAGCCCTCGCCGCCCAGTGCCCTTC
GGCCTCGGGGGCGGGCGCCTGCGTCGGTCTCCGCGAAGCGGGAAAGCGCGGCGGCCGCCGGGATTCGGGCGCCGCGGCA
GCTGCTCCGGCTGCCGGCCGGCGGCCCCGCGCTCGCCCGCCCCGCTTCCGCCCGCTGTCCTGCTGCACGAACCCTTCCAAC
TCTCCTTTCCTCCCCCACCCTTGAGTTACCCCTCTGTCTTTCCTGCTGTTGCGCGGGTGCTCCCACAGCGGAGCGGAGATTA
CAGAGCCGCCGGGATG

* Searching results of EGR1 and SP1 binding regions downstream of TCACA by Genomatix software

EGR-1 (-): 5’ gggtgAGGGggagtcggcg 3’

Sp1 (-): 5’ aggcGGGGggtgagggg 3’

EGR/SP1 overlapping region in LEF1: CCCCTCACCC

EGR1

Common Match Table (click on headers to sort columns)

#	Seq. name	Gene Id	Symbol	Family	Matrix	Start	End	Strand	Matrix sim.	Core sim.	Sequence
20	TempSeq_imNCtpwt			V$EGRF	V$WT1.01	79	97	-	0.928	0.837	gggtgAGGGggagtcggcg

SP1

Transcription Factor Binding Sites common to at least 100% of the sequences

Graphical View | Match Summary Table | Common Matches

Common Match Table (click on headers to sort columns)

#	Seq. name	Gene Id	Symbol	Family	Matrix	Start	End	Strand	Matrix sim.	Core sim.	Sequence
28	TempSeq_imNCtpwt			V$SP1F	V$TIEG.01	88	104	-	0.849	1.000	aggcGGGGggtgagggg

The distal promoter regions of MMP9 (A), ZEB1 (B), fibronectin (C) and lymphoid enhancer-binding factor 1 (LEF1) (D) upstream of translational initiation codon (marked as ATG) contained similar sequence architecture including TCACA (marked with yellow) and downstream EGR1and SP1 binding region (marked with light blue and red). The EGR1and SP1overlapping region in promoters of MMP9 (A), fibronectin (C) and LEF1(D) are marked with green. There are two separated region (by 6 bp) of alternative EGR1and SP1 binding site on ZEB1 promoter. The binding sequences for EGR1 and SP1 for each promoter are demonstrated in the table quoted from the searching results obtained by Genomatix software. TPA-responsive elements in MMP9 and ZEB1 promoters, located at (-832 ~ -771 bp) and (-1061~-830), respectively, are marked with

Supplemental Fig. 2 MAP for ChIP fragment of MMP9 / ZEB1 promoter and EMSA probe for MMP9 promoter

(A)

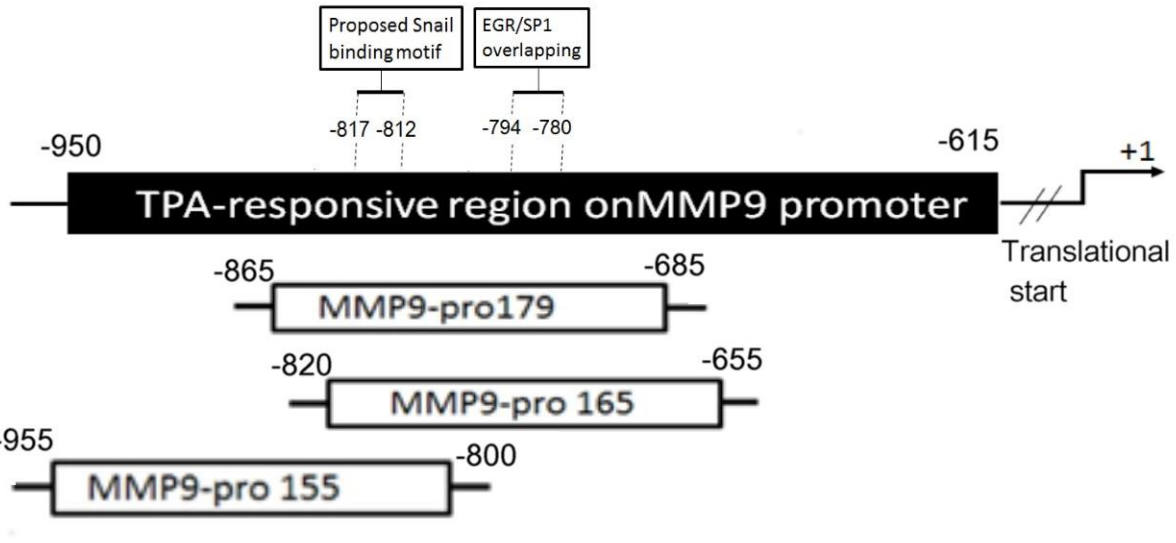

(B)

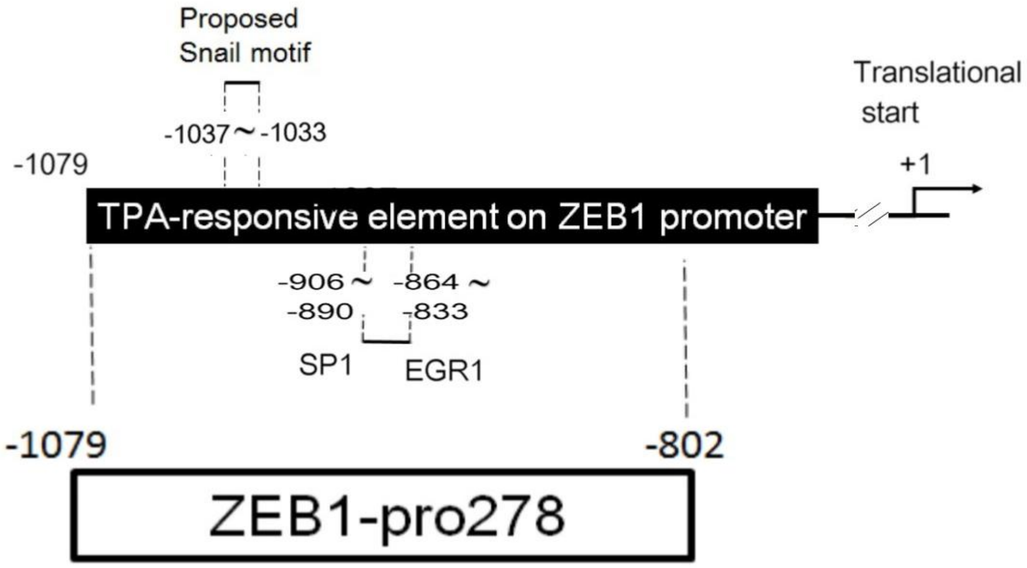

(C)

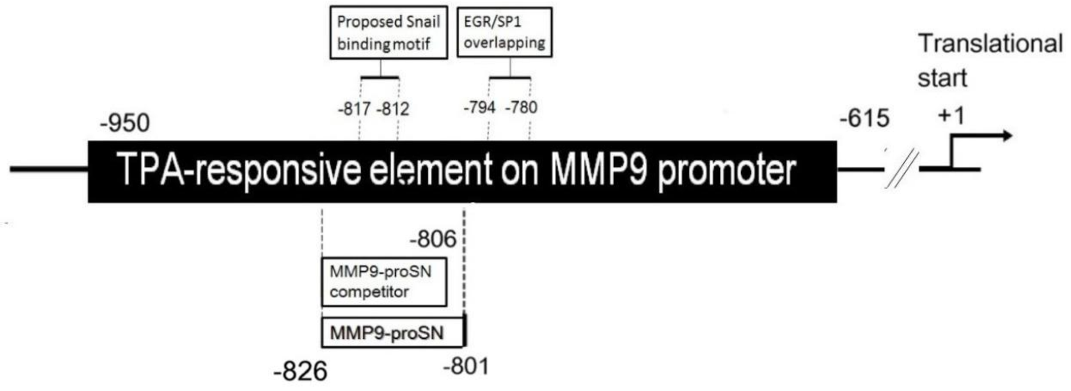

Schematic MAP showing the PCR fragment amplified for the ChIP assay of Snail, EGR and SP1 on MMP9 (A) and ZEB1 (B) promoters and EMSA probe (C) for MMP9 promoter. The black bars represent the distal promoter region of MMP9 (A,C) and ZEB1 (B) from -950 bp and -1079 bp, respectively, upstream of the translation start site. In (A), the sequence of MMP9-pro179 and MMP9-pro165 are located between -865 to -685 bp and -820 to -655 bp, respectively containing both the proposed Snail target region and EGR/SP1 overlapping binding site, whereas MMP9-pro 155 is located between -955 to -800 bp containing the proposed Snail target region only. In (B) , the sequence of ZEB1 pro278 are located between -1079 bp to -802 containing both the proposed Snail target region and EGR/SP1 overlapping binding site. In (C), the EMSA probe of MMP9, MMP9-proSN, containing the sequence

from -817 to -794 bp spanning the region of proposed Snail motif but not EGR/SP1 binding site.

Supplemental Fig. 3 (S3 Fig) Deletion or mutation of the proposed Snail binding motif decreased TPA-induced promoter activation of MMP9 and in a similar extent

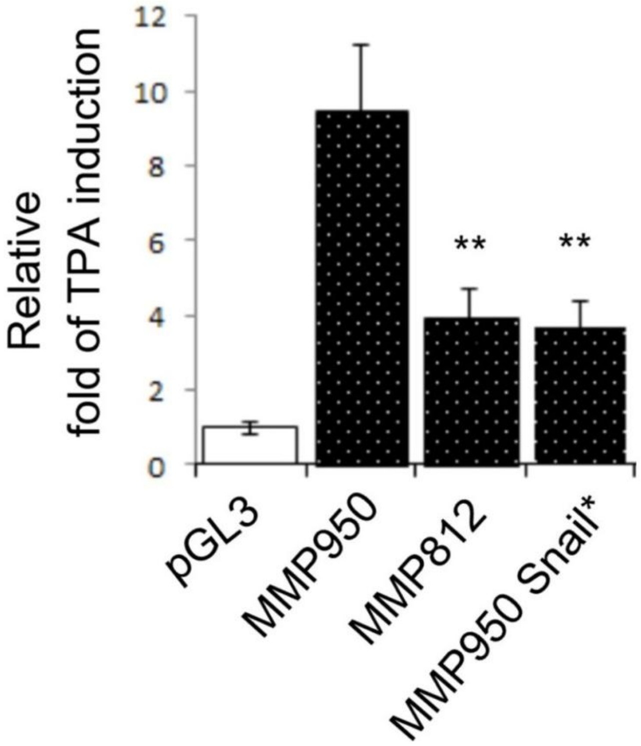

HepG2 cells were transfected with pGL3 vector, the indicated MMP9 promoter plasmids including full length promoter MMP9-950, MMP9-812 with deletion of Snail binding motif, or mutant promoter MMP9-950 Snail* with alteration on the putative binding region of Snail transcriptional factors, for 24 h. Subsequently, the cells were untreated or treated with 50 nM TPA for 24h and then single luciferase assay were performed. The relative fold of TPA induction for each promoter were quantitated as the activity of TPA treated *vs* untreated, taking the data of pGL3 as 1.0. (**) represent the statistical significant difference ($p<0.05$, N=3) of fold of TPA induction between each of the indicated promoters and the full length promoter (MMP9-950) .

Supplemental Fig. 4 (S4 Fig) Actinomycin D affected TPA-induced EGR and Snail expression

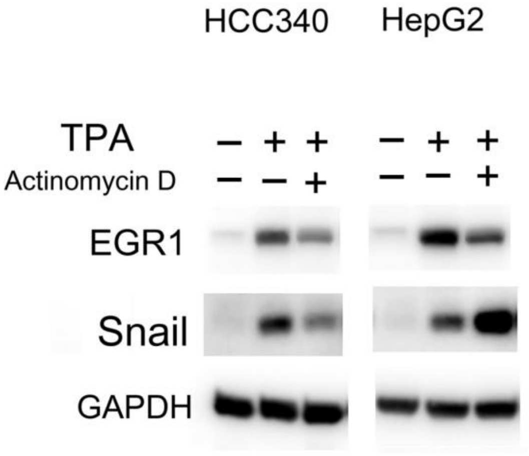

HepG2 and HCC340 cells were untreated, treated with TPA (50 nM) alone or TPA coupled with actinomycin D (20 nM) for 4 h. Western blot of indicated molecules were performed, using GAPDH as a internal control. The data is representative of two reproducible experiments.

Supplemental Fig. 5 (S5 Fig) TPA-induced mRNA expression of fibronectin and lymphoid enhancer-binding factor (LEF**)**

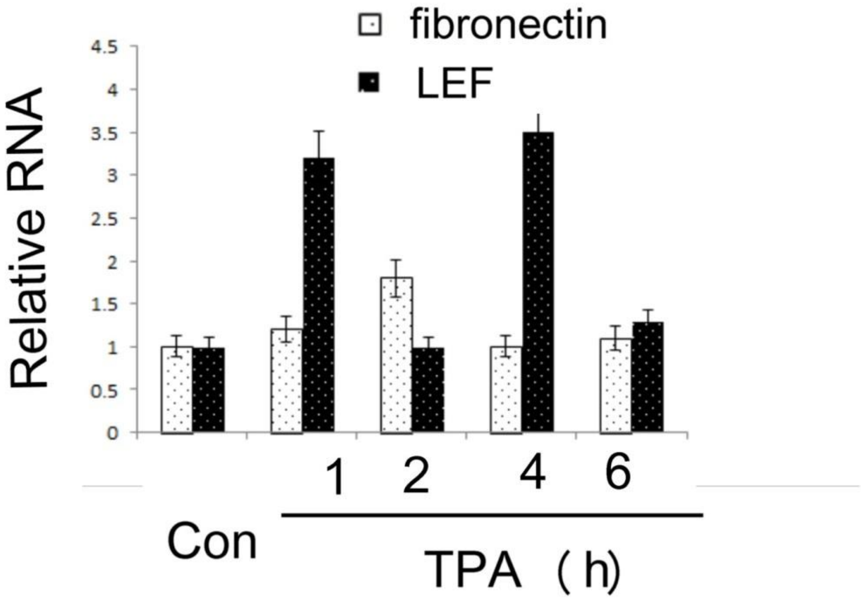

HepG2 cells were treated with 50 nM TPA for the time indicated. Quantitative RT-PCR of fibronectin and LEF were performed. The data shown are average of two reproducible experiment with C.V. 12%

Supplemental Fig. 6 (S6 Fig) TPA induced slight association of SP1 with Snail

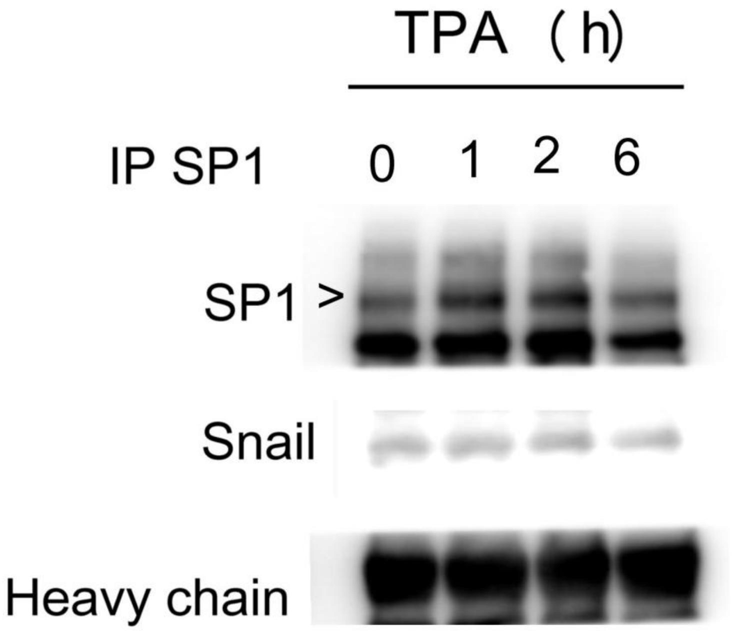

HepG2 cells were treated with TPA (50 nM) for indicated time. Immunoprecipitation (IP) of SP1 followed by Western blot of SP1 and Snail were performed, using heavy chain of Ab as an internal control. The data is representative of two reproducible experiments.
